# Disease and freeways drive genetic change in urban bobcat populations

**DOI:** 10.1111/eva.12226

**Published:** 2014-12-02

**Authors:** Laurel E K Serieys, Amanda Lea, John P Pollinger, Seth P D Riley, Robert K Wayne

**Affiliations:** 1 Department of Ecology and Evolutionary Biology, University of CaliforniaLos Angeles, CA, USA; 2 Department of Biology, Duke UniversityDurham, NC, USA; 3 Santa Monica Mountains National Recreation Area, National Park ServiceThousand Oaks, CA, USA

**Keywords:** balancing selection, disease, freeways, immune-linked loci, major histocompatibility complex, population structure, Toll-like receptors, urbanization

## Abstract

Urbanization profoundly impacts animal populations by causing isolation, increased susceptibility to disease, and exposure to toxicants. Genetic effects include reduced effective population size, increased population substructure, and decreased adaptive potential. We investigated the influence that urbanization and a disease epizootic had on the population genetics of bobcats (*Lynx rufus*) distributed across a highly fragmented urban landscape. We genotyped more than 300 bobcats, sampled from 1996 to 2012, for variation at nine neutral and seven immune gene-linked microsatellite loci. We found that two freeways are significant barriers to gene flow. Further, a 3-year disease epizootic, associated with secondary anticoagulant rodenticide exposure, caused a population bottleneck that led to significant genetic differentiation between pre- and post-disease populations that was greater than that between populations separated by major freeways for >60 years. However, balancing selection acted on immune-linked loci during the epizootic, maintaining variation at functional regions. Conservation assessments need to assay loci that are potentially under selection to better preserve the adaptive potential of populations at the urban–wildland interface. Further, interconnected regions that contain appropriate habitat for wildlife will be critical to the long-term viability of animal populations in urban landscapes.

## Introduction

Anthropogenic barriers reduce habitat connectivity, impeding gene flow between populations and accelerating the loss of genetic diversity due to drift (Epps et al. 2005; Hedrick 2005; Riley et al. 2006; Keyghobadi 2007; Delaney et al. 2010; Lee et al. 2012). Urbanization may also contribute to increased disease exposure (Bradley and Altizer 2007) that can cause precipitous population declines (LoGiudice 2003; Riley et al. 2007). Genetic consequences that accompany reduced population size and connectivity include inbreeding depression that may increase the probability of population extinction (Saccheri et al. 1998; Coltman et al. 1999; Keller 2002; Spielman et al. 2004) and the loss of adaptive potential that reduces the ability of populations to respond to novel selection regimes (Lande 1998; Frankham 2005; Keyghobadi 2007). Yet, despite recognition that urban populations are vulnerable to reduced genetic connectivity and population declines, few studies have characterized neutral and adaptively relevant genetic diversity in urban systems (Hitchings and Beebee 1997; Keyghobadi 2007).

Gene regions related to immune function are considered paradigms for the study of important adaptive genetic diversity (Piertney and Oliver 2005). The major histocompatibility complex (MHC) and Toll-like receptors (TLRs) encode key components of the vertebrate immune system and are among the most polymorphic families of genes known (Klein 1986; Jepson et al. 1997; Hill 1998; Roach et al. 2005; Iwasaki and Medzhitov 2010). TLRs are a critical component of innate immunity that provide nonspecific protection against a variety of disease-causing organisms (Aderem and Ulevitch 2000; Janeway and Medzhitov 2002). MHC genes encode cell surface molecules that present antigens to T lymphocytes to initiate innate and adaptive immune responses (Klein 1986). Positive and balancing selection are considered important drivers of genetic diversity at these regions (Bernatchez and Landry 2003; Piertney and Oliver 2005; Ferrer-Admetlla et al. 2008; Areal et al. 2011) although studies in free-ranging populations have implicated both drift and selection in shaping variation at immune genes (Bernatchez and Landry 2003; Piertney and Oliver 2005; Bollmer et al. 2011).

**Figure 1:**
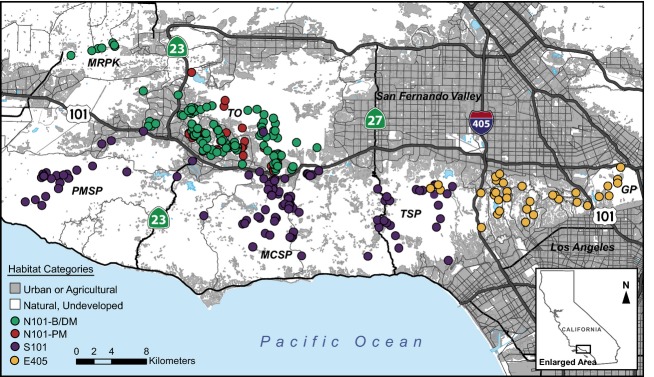
Map of Santa Monica Mountains National Recreation Area where bobcats were sampled. Colored circles represent individual bobcat sampling locations, and colors correspond with the predominant structure cluster assignment for each individual. Major freeways of interest include the 101 Freeway and the I-405. The 23 (south of the 101 Freeway) and the 27 are secondary roads that intersect the study area. Specific locations referenced in the study: MRPK, Moorpark satellite population; TO, Thousand Oaks; PMSP, Point Mugu State Park; MCSP, Malibu Creek State Park; TSP, Topanga State Park; and GP, Griffith Park. N101-B/DM: N101 before/during mange; N101-PM: N101 postmange.

To address the consequences of urbanization on wildlife population genetics, we focused on a well-studied population of bobcats (*Lynx rufus*) living in and around Santa Monica Mountains National Recreation Area (SMMNRA). This region comprises a collection of protected park areas near downtown Los Angeles. Bobcats inhabiting SMMNRA have been monitored by National Park Service (NPS) biologists since 1996. Within a localized region of SMMNRA, the NPS has demonstrated that a major freeway (US-101, Fig.1) acts not only as a barrier to movement for bobcat and coyote (*Canis latrans*) populations, but potentially also as a social barrier. Specifically, the small percentage of bobcats (11.5%) and coyotes (4.5%) that successfully crossed the 101 Freeway were unlikely to establish territories and reproduce (Riley et al. 2006). As a result, carnivore populations separated by the US-101 were significantly genetically differentiated, although they were separated by <1 km (Riley et al. 2006). The 101 Freeway has also been shown to be an important barrier to gene flow for other species including side-blotched lizards (*Ula stansburiana*), western fence lizards (*Sceloporus occidentalis*), western skinks (*Plestiodon skiltonianus*), and wrentits (*Chamaea fasciata*) (Delaney et al. 2010). Taken together, these findings suggest that major freeways can severely interrupt gene flow in a variety of taxa.

Urbanization has also negatively impacted SMMNRA bobcat populations through increased disease susceptibility. From 2002 to 2005, a notoedric mange epizootic associated with secondary anticoagulant rat poison exposure was the greatest source of mortality for bobcats (Riley et al. 2007). During this period, the annual survival rate for radio-collared animals fell by >50% and in 2003 the mange mortality rate reached a high of 51%. Long-term samples were collected from this population from 1996 to 2012, allowing the rare opportunity to make direct comparisons before, during, and after the population decline. Furthermore, samples were more recently collected throughout SMMNRA from populations experiencing varying levels of habitat fragmentation and isolation, allowing a determination of how barriers to gene flow (e.g., major freeways) accelerated the genetic consequences of a population decline associated with disease.

As a result, this population is appropriate for studying the increasingly widespread impacts of urbanization on free-ranging animal populations, the influence of habitat fragmentation on gene flow and neutral genetic diversity, and the effects of increased disease susceptibility and a consequent population decline on immune-related genetic variation.

We genotyped 365 bobcats at nine neutral microsatellite loci and 299 bobcats at seven microsatellite loci in or near immune-related genes. We investigated how freeways and a population decline associated with disease influence population isolation, structure, and differentiation. We also characterized the role of selection as a result of disease and genetic drift on immune-related genes in populations separated by urbanization and freeways. Our use of both neutral and immune-linked microsatellite loci enabled us to determine whether habitat fragmentation, urbanization, and the disease outbreak and subsequent population decline differentially affect functional versus neutral genetic diversity.

We predict that major urban barriers (e.g., freeways) impede gene flow and promote population isolation and genetic drift; consequently, populations separated by freeways should be highly differentiated at neutral loci. In addition, we hypothesize that the population decline associated with the disease outbreak should enhance genetic drift; thus, samples collected after the disease outbreak are expected to show reduced genetic diversity at neutral loci compared to samples collected before the disease outbreak and to be genetically differentiated from each other. With respect to immune-related loci, we expect that heterogeneity in disease prevalence and in the severity of population isolation would lead to differential patterns of selection and genetic drift. Specifically, we predict that the population severely impacted by disease would experience directional or balancing selection at immunologically important genes, as genetic variation at these loci may confer resistance to disease (Schröder and Schumann 2005; Froeschke and Sommer 2005; Barreiro et al. 2009, Savage and Zamudio 2011; de Assunção-Franco et al. 2012). Balancing selection would be expected to maintain or enhance variation across a bottleneck whereas directional selection would reduce genetic diversity. However, for populations with a high degree of isolation or unaffected by disease, we predict that genetic drift may be the critical driver of genetic variation at immune-linked loci.

## Materials and methods

Bobcats were sampled in and around Santa Monica Mountains National Recreation Area (SMMNRA, Fig.1) in southern California in the United States. The study area is comprised of more than 1264 km^2^, encompassing large regions of continuous habitat with minimal urban development (Fig.1, PMSP, MCSP, and TSP), as well as highly fragmented areas that extend east through Los Angeles (Fig.1, areas east of the I-405). Approximately 70% of the study area is comprised of undeveloped parks and natural open spaces that are considered suitable habitat for bobcats. The area straddles two major 8- to 10-lane freeways, US-route 101 and Interstate-405. US-route 101 was established in 1949 with an average of 321 000 vehicles daily (www.fhwa.dot.gov). Route I-405, established in 1962 (www.cahighways.org), is the most travelled highway in the country with an average of 374 000 vehicles daily (www.fhwa.dot.gov). Many secondary roads are also found throughout SMMNRA. Human land uses within SMMNRA include commercial and residential development.

Bobcats sampled from SMMNRA were classified as belonging to one of the following three geographic regions: (i) highly fragmented habitat east of I-405 and including an area east of the 101 Freeway in Griffith Park (GP) sampled from 2010 to 2011 (E405 population); (ii) a region west of the I-405 and south of the 101 Freeway (S101 population) that is comprised largely undeveloped protected parkland intersected by secondary roads and pockets of urban development; and (iii) an area north of the 101 Freeway (N101 population) consisting largely of habitat patches interspersed with roads and development (Fig.1).

From 1996 to 2012, long-term bobcat sampling occurred in the N101 region, as well as in Malibu Creek State Park (Fig.1, MCSP), an isolated subset of S101 as previously described (Riley et al. 2006). From 2002 to 2005, an epizootic of notoedric mange associated with secondary anticoagulant rodenticide exposure occurred primarily north of the 101 Freeway, causing a sharp population decline during the latter half of the epizootic (Riley et al. 2007). At the peak of the mange epizootic, the annual survival rate declined from >75% to <30% (Riley et al. 2007), and complete extirpation of bobcats occurred in some N101 habitat patches (Riley et al. 2014). Further, scat surveys conducted on transects in the N101 region revealed a decline of approximately 90% of bobcat scat collected in 2001 compared with 2005 (Riley et al. 2007; NPS unpublished data), providing additional support that the N101 population suffered an extreme population reduction. Bobcats were sampled before, throughout, and after the mange epizootic. Consequently, analyses described below were primarily conducted on two N101 groups that we predict represent separate populations. We use the ecological definition of a population as comprising a group of individuals that are the same species that exist in a particular time and space (Krebs 1994). The first population included animals sampled before and during the mange epizootic (N101 before/during mange) from 1996 to 2005. These two periods are grouped because we expect genetic consequences of the disease outbreak to occur after the population decline that primarily occurred during 2003–2005. The second population consisted of animals sampled after the mange epizootic (N101 postmange) from 2006 to 2012. Thus, a total of four putative populations for this study include E405, S101, N101 before/during mange and N101 postmange.

Sample collection across all regions consisted of blood, tissue or buccal swabs obtained by capturing animals or opportunistic tissue obtained postmortem from carcasses discovered in the study area (Table S1). Fresh scat samples were opportunistically collected during trapping seasons. Bobcats were captured with padded foothold traps (1996–1998) and cage and box traps (2000–2012) as previously described (Riley et al. 2006). Once captured, animals were aged, sexed, weighed, measured, ear-tagged, and a subset of individuals were radio-collared as part of ongoing NPS research. All animals were then released at the capture site. Animal capture, handling, and sample collection protocols were approved by the Office of Animal Research Oversight of the University of California, Los Angeles (Protocol ARC#2007-167-12). Protocols underwent extensive review to minimize animal stress and suffering. Scientific collecting permits were authorized through California Department of Fish and Wildlife (SC-9791).

### Genotyping

DNA was extracted from tissue, blood, and buccal swabs using the QIAamp® DNA Mini Kits (Qiagen, Germantown, MD, USA) and from fecal samples using QIAamp® DNA Stool Mini Kits (Qiagen) according to the manufacturer's protocols. Individuals were genotyped using nine supposed neutral dinucleotide microsatellite markers (FCA008, FCA023, FCA026, FCA043, FCA045, FCA077, FCA090, FCA096, and FCA132) previously used in bobcat studies (Ernest et al. 2000; Riley et al. 2006). To ensure accurate genotypes of lower quality fecal DNA and to prevent contamination with higher quality tissue DNA, polymerase chain reactions (PCRs) for scat samples were performed on 96-well plates separately from those used to genotype tissue samples. To ensure accurate genotype calls, fecal samples were genotyped multiple times. Heterozygous loci were confirmed with at least one additional amplification, and homozygous loci were amplified a minimum of three times. When consistent results could not be achieved for specific loci after multiple amplifications, the single-locus genotypes were treated as missing data. Any scat samples with more than two loci of missing data were excluded from analyses.

To assess the influence of urbanization and disease on patterns of genetic diversity at regions of functional importance, samples were also genotyped at seven immune-related microsatellite loci. We chose five MHC regions found on chromosome B2 in the domestic cat (Beck et al. 2005) that included a Class I locus (FLA1) and four Class II loci (DRA1, DRB1, DRB3, and DRB4). Additionally, two Toll-like-receptor-associated loci, found on chromosomes B1 and D4, were chosen (TLR3 and TLR4) using the cat genome assembly version Felcat4 (Pontius et al. 2007).

We used msatcommander (Faircloth 2008) to design primers (Table S2) for the amplification of immune regions. The program enables rapid microsatellite repeat detection based on motif-matching with a specific, user-defined DNA sequence. The program also allows rapid and automated design of locus-specific primers and 5′-tailing primers. Primers designed using this program were validated with domestic cat DNA and tested for polymorphism and amplification efficacy with bobcat DNA. Patterns consistent with a single locus were observed in all amplifications.

Genotypes were obtained by PCR amplification of all 16 loci as in Riley et al. (2006). Established PCR conditions were used for the hybrid combination primer (a two-step cycle; Boutin-Ganache et al. 2001). Primer dye-labeling utilized BeckmanCoulter dye D4, and PCR products were sized on the BeckmanCoulter CEQ2000XL DNA Analysis System (Riley et al. 2006).

### Validating and characterizing microsatellite data

For each neutral and immune-linked locus, deviations from Hardy–Weinberg equilibrium (HWE) and linkage disequilibrium (LD) were tested using genepop version 4.0 (Raymond and Rousset 1995). Each marker pair was tested for LD within each population and across all populations. Because >100 comparisons were made, we used the Bonferroni method to correct the critical value corresponding to *α *= 0.05 for 104 comparisons (*α *= 0.0005) (Rice 1989). Global tests of heterozygote deficit and excess were conducted for each population, as well as for each locus within populations, and because 32 tests were conducted per population, we corrected for multiple tests (*α *= 0.002). Genotyping error, the presence of null alleles, scoring errors, and allele dropout were evaluated using micro-checker (van Oosterhout and Hutchinson 2004).

### Genetic diversity

Observed (*H*_O_) and expected (*H*_E_) heterozygosities (Nei 1987) were calculated using cervus (Marshall et al. 1998). Per-locus allelic richness (*A*_R_) (Mousadik and Petit 1996) and the inbreeding coefficient (*F*_IS_) were estimated using fstat 2.9.3 (Goudet 1995). We estimated *F*_IS_ significance for all loci and all populations using 560 randomizations in fstat 2.9.3. Data were tested for normality using the Shapiro–Wilk test. Across population differences for each measure were tested either with a Kruskal–Wallis or with an anova. Pairwise comparisons were calculated with Mann–Whitney or *t*-tests (*α *= 0.05). Because theory predicts changes in heterozygosity when a significant population decline occurs, allelic richness, observed and expected heterozygosity, and the inbreeding coefficient were also tested for differences between the N101 before mange (1996–2001) and the N101 postmange (2006–2012). Genetic diversity values for the N101 population before, during, and after the mange epizootic are presented in the Supporting Information (Table S3). The significance of differences was tested with Wilcoxon rank sum or *t*-tests (*α *= 0.05). All across-group and pairwise analyses were conducted in r (R Development Core Team 2010).

### Demography

We obtained estimates of effective population size (*N*_e_) across populations using a LD method (Waples 2006) implemented in LDNe (Waples and Do 2008). To evaluate the fine-scale temporal impact of the disease outbreak on the effective population size over time, *N*_e_ for the N101 population was measured in 1- to 4-year increments, depending on the number of animals captured yearly such that all *N* > 20 (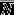 = 26; Table1). The program is reported to perform well in nonideal populations. For example, Waples (2006) found that for smaller sample sizes (*N* = 10 or 20), the performance of the program was nearly equivalent to that for sample sizes of *N* ≥ 30.

**Table 1:** Effective population size (*N*_e_) and 95% parametric confidence intervals for bobcat populations in SMMNRA. For the N101 population affected by the notoedric mange epizootic, *N*_e_ is partitioned by sampling years

Population	Year	*N*	*N*_e_	95% CI
N101	1996–1998	33	47.2	25.9–130.7
	2000–2001	22	17.6	12.1–27.6
	2002*	22	14.3	9.9–21.8
	2003*	25	14.6	10.8–20.1
	2004–2005*	22	8.6	5.6–13.0
	2006–2008	20	13.3	8.3–22.9
	2009–2012	30	14.5	9.4 – 23.0
S101	1996–2012	126	97.7	71.0–143.6
E405	2010–2011	47	34.4	21.8–61.2

SMMNRA, Santa Monica Mountains National Recreation Area.

* Years during which the mange epizootic occurred.

We used two methods to test for evidence of a genetic bottleneck across all sampled populations and years. Using the neutral loci, we employed bottleneck 1.2.02 (Cornuet and Luikart 1996; Piry et al. 1999) to test for significant heterozygosity excess compared to mutation-drift equilibrium expectations for a stable population. As above, we divided N101 samples into 1- to 4-year increments (all *N* > 20, 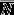
* *= 26). Cornuet and Luikart (1996) suggest a minimum sample size of 20 diploid individuals to achieve a reasonably high degree of power in bottleneck tests. We tested each subset for heterozygosity excess. For the MCSP population subset of S101, we also tested for heterozygosity excess during 1996–2001 (*N* = 30) and 2006–2012 (*N* = 26) to determine whether a population bottleneck occurred regionally across the study area. We ran 1000 iterations of the two-phase model (TPM) recommended for microsatellite data, with a variance (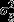 ) of 12% and 90% single-step mutations (*p*_s_* *= 90%) (Rienzo 1994; Brinkmann et al. 1998; Piry et al. 1999; Garza and Williamson 2001). Recently, Peery et al. (2012) suggested experimenting with alternative parameters, and specifically testing *p*_s_* *= 78%, because this value represents the mean estimate of single-step mutations in vertebrate studies. Thus, we also tested for heterozygote excess using this alternative parameter. To evaluate significance, we used the Wilcoxon signed rank test recommended for polymorphic microsatellite data (Piry et al. 1999).

In addition, we tested for a bottleneck signature using Garza and Williamson's *M*-ratio method implemented in the program m_p_val (Garza and Williamson 2001). The approach calculates *M*, the mean ratio of the number of alleles to range in allele size, which can be used to detect reductions in effective population size. In this case, *M* ratios will be smaller than 1.0, and for populations known to have undergone a significant decline in effective population size, the approximate value of *M* is ≤0.70 (Garza and Williamson 2001). We used the parameters suggested by Garza and Williamson for the proportion of single-step mutations (*p*_s_* *= 90%) and the average size of multistep mutations (Δ_g_* *= 3.5). As above, we also tested *p*_s_* *= 78%. Garza and Williamson (2001) recommend that sampling approximately 25 diploid individuals is sufficient for most vertebrate populations, although they indicate that there are no clear guidelines for determining an adequate sample size. We tested four critical values of *θ* (4*N*_e_μ) based on a range of effective population size estimates calculated for bobcat populations in the study area using LDNe as described above. These values corresponded with a *N*_e_ of 25 (*θ *= 0.05), 50 (*θ *= 0.1), and 150 (*θ *= 0.3), with a fixed mutation rate (μ) of 5 × 10^−4^ per locus per generation. Statistical significance of the *M*-ratio was evaluated with 10 000 iterations.

### Population structure and genetic differentiation

We investigated the number of genetic clusters and estimated levels of population differentiation separately for neutral and immune-linked loci using two Bayesian clustering methods. structure 2.3.4 infers population assignments without any *a priori* assumptions about sample location (Pritchard et al. 2000). We also used Geneland 4.0.3 that utilized sample locations and genotype data in a spatial statistical model to infer the number of genetic clusters (*K*), as well as the spatial distribution of, and genetic discontinuities between, assigned populations (Guillot et al. 2005).

**Figure 2:**
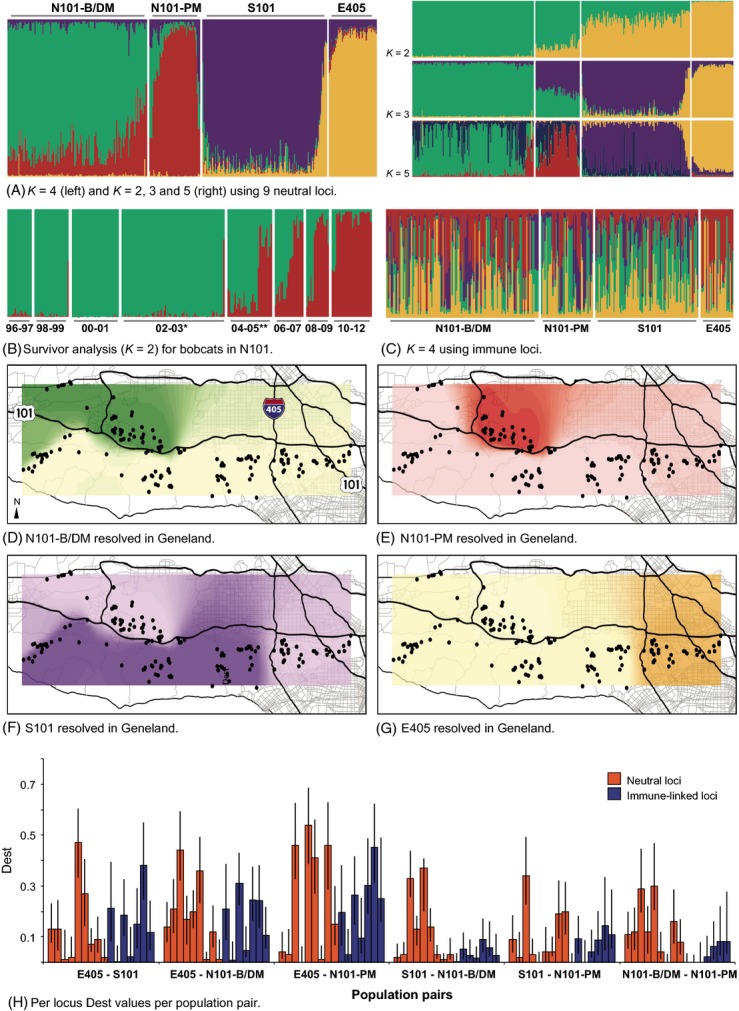
Results of population structure analyses, using capture location as prior information, using neutral loci (A, B, D–G) and immune-linked loci (C). For structure plots (A–C), each vertical bar represents one individual. The shading of each bar corresponds to the probability of genetic assignment to one of four populations of bobcats that included N101 before/during mange (N101-B/DM, green), N101 postmange (N101-PM, red), S101 (purple), or E405 (gold). (A) *K *=* *2–5 results for bobcats across the study area. (B) Analysis (*K *=* *2) using survival information for individuals in the N101 population from 1996 to 2012. *Indicates when mange entered the population, **Indicates when a genetic bottleneck is detected. (C) *K *=* *4 results for bobcats across the study area using immune-linked loci. No distinguishable structure was present for any *K*-value. (D–G) Geneland results, interpolated over Santa Monica Mountains National Recreation Area (SMMNRA), based on runs with migrants removed. Black circles represent bobcat sampling locations. Color assignments correspond with structure results. Darker colors represent a high probability of assignment to a focal population, while the lighter color represents a high probability of assignment to any other population. (D) N101 before/during mange resolved (green). (E) N101 postmange resolved (red). (F) S101 resolved (purple). (G) E405 resolved (gold). Admixture between N101 and S101 resulted in unclear population assignment boundaries in the westernmost region of SMMNRA in (D) and (F). (H) Per-locus *D*_est_ values for all population pairs. A significant difference between the overall neutral and immune *D*_est_ values was present only for the N101-B/DM–N101-PM population pair.

Using structure, we first inferred the number of genetic clusters (*K*) without any *a priori* assumptions about the sample location. The stability of the inferred clusters was evaluated using 10 independent runs at *K *=* *1–10 with a burn-in period of 50 000 iterations followed by 500 000 MCMC cycles. We examined both raw probability values of LnP(*K*) and the Δ*K* estimate (Evanno et al. 2005) using structure harvester (Earl and vonHoldt 2011) to help evaluate the most likely number of genetic clusters. We used clumpp 1.1 (Jakobsson and Rosenberg 2007) to align the multiple structure replicates. Because the most likely population clustering corresponded strongly with our geographical sampling (see results), we performed analyses to evaluate the individual cluster assignments using capture location as prior information, as previously described (Riley et al. 2006), and we report these results (Figs2A–C, S1A–C and S2A–D, and Table S4).

structure was also used to examine how disease affected population turnover in the mange-affected population. Using yearly survival data collected during ongoing NPS radio-telemetry studies in the N101 area (Riley et al. 2003, 2010; NPS unpublished data), we grouped individuals into two-year increments, corresponding with the two-year generation time estimated for bobcats (Knick et al. 1985). Each time increment included all animals that were known to be alive during that time period. If individuals were alive for more than two years, they were represented in each time increment they were known to be alive. Using this modified dataset, we performed structure analyses as described above.

Finally, we used structure to perform assignment tests to identify potential migrants within the dataset. Genetic assignment tests can identify the population of origin for each individual, and individuals that are assigned to a population different than the one in which they were captured are considered potential migrants (Berry et al. 2004). We modified the structure input data file to reassign individuals to their putative population of genetic origin based on the cluster assignment results and then calculated the posterior probability of correct population assignment as previously described (Riley et al. 2006). Individuals were considered migrants if their assignment probabilities and posterior probabilities were >50% to a population different than the one in which they were captured.

We used Geneland (4.0.3; Guillot et al. 2005) to examine spatial and temporal patterns of genetic discontinuities across the landscape. Because genotypes used by the program are georeferenced, we created two datasets to measure the potential temporal difference in population structure across samples collected over the long term. In addition to examining potential anthropogenic features that were related to genetic discontinuities, we were also interested in whether the disease outbreak influenced genetic discontinuities. Therefore, we assessed two datasets that included the following: (i) all bobcats sampled from 2006 to 2012 [E405, S101, and N101 postmange (Fig.1)]; and (ii) bobcats sampled from E405 to S101 during 2006–2012 and bobcats sampled in N101 premange (1996–2001). In both datasets, we also included 10 individuals from the Moorpark satellite population (MRPK, Fig.1) sampled from 2010 to 2011 to determine which N101 population they assigned to. For each dataset, we ran 500 000 iterations with a thinning of 100 and *K *=* *1–10. This test was carried out five times with each dataset to evaluate the stability of the most likely *K*. Migrants could influence genetic discontinuities along the landscape as inferred by Geneland. Therefore, we tested the above datasets with (i) all individuals; and (ii) without the observed four migrants identified by structure (see Results).

Finally, to examine the impact of disease and urban development on genetic differentiation, we estimated pairwise *F*_ST_ using fstat 2.9.3.2 (Goudet 1995). The use of *F*_ST_, however, has been recently criticized (Jost 2008; Heller and Siegismund 2009), and so we also calculated an alternative estimator of population differentiation, *D*_est_ (Jost 2008) using the program smogd (Crawford 2010). *D*_est_ more accurately accounts for differences in allelic diversity than *F*_ST_ particularly for highly polymorphic microsatellite markers (Heller and Siegismund 2009). *D*_est_ was calculated with 1000 bootstraps for each marker class. To test the significance of overall *D*_est_ values for neutral and immune-linked loci across all populations, and the per-immune locus values in the N101 populations impacted by disease, we computed probabilities using 999 permutations in GenAlex 6.5 (Peakall and Smouse 2012). For comparison to previous publications, we provide between population *F*_ST_ values in the supplemental information (Table S5).

### Relatedness

We calculated individual pairwise relatedness (R) using the program Maximum-likelihood (ml)-Relate (Kalinowski et al. 2006) for each population using neutral loci. We calculated the mean relatedness per population and compared the mean relatedness across populations using an anova, and between population pairs using *t*-tests.

### Tests for selection

We evaluated the role of selection in shaping immune-linked loci variation. We used per-locus *D*_est_ for each of the total 16 neutral and immune-linked microsatellite loci and calculated pairwise comparisons between the two classes of markers for each population pair using Mann–Whitney tests. By comparing the level of divergence based on the neutral and immune classes of markers, it may be possible to detect whether selection (balancing or directional) or drift dominates the observed patterns of variation at immune regions (Bernatchez and Landry 2003; Piertney and Oliver 2005). Specifically, population divergence should be less for markers under balancing selection, in comparison with neutral markers, because of the greater effective migration rate of alleles under balancing selection (Scheierup et al. 2000). In contrast, if selection differentially favors particular immune alleles among different environments and populations, theory predicts that population divergence will be greater at immune markers than neutral markers (Bernatchez and Landry 2003; Piertney and Oliver 2005).

Additionally, for each population pair, we evaluated the deviation from neutrality for all immune and neutral loci using Beaumont and Nichol's Fdist2 method implemented in lositan (Beaumont and Nichols 1996; Antao et al. 2008). This approach evaluates the relationship between *F*_ST_ and expected heterozygosity under an island model of migration with neutral markers. Outlier loci that have excessively high or low *F*_ST_ compared to neutral expectations are considered potentially under selection. For each population pair, we carried out 100 000 simulations using composite neutral and immune genotypes, assuming a stepwise mutation model.

## Results

### Bobcat sampling

We obtained 287 blood and tissue samples from live-captured bobcats from four putative bobcat populations (E405, S101, N101 before/during mange, and N101 postmange) during the period 1996–2012. Opportunistic samples were collected postmortem from 39 mortalities from 1996 to 2012, and 39 scats were collected from 2008 to 2012. A total of 365 samples (live-trapped, mortalities, and scat) were genotyped for nine neutral loci, and 299 individuals were genotyped for seven immune-linked loci (live-trapped or sampled postmortem, Table S1). Of samples genotyped at the neutral loci, 3% had data missing for one locus, and one scat had data missing for two loci. Of samples typed for the seven immune-linked loci, 3% had data missing for one locus, and no samples were missing data for more than one locus (Table S6). No genotyping errors or allele dropout was observed. Five neutral and six immune-linked loci showed evidence of null alleles, although no loci exhibited evidence of null alleles across all four populations. In at least one of the four populations, four loci (FLA1 and FCA045 in S101, FCA090 and FCA096 in E405, and FCA090 in N101 postmange; Tables S7 and S8) exhibited evidence of null alleles with a frequency of >0.10 across the four methods implemented in micro-checker. The high estimates of null alleles could lead to an overestimation of genetic differentiation between populations (Chapuis and Estoup 2007; Carlsson 2008). However, true null alleles are generally only found in populations with large effective population sizes (e.g., *N*_e_ ≥ 50 000), and if the genotyped population is not completely representative of the focal population, null allele frequencies may be artificially inflated (Chapuis and Estoup 2007). Given the cryptic nature of the bobcat, complete population sampling would not be feasible. The evidence of null alleles in our study may be due to the presence of actual null alleles in some of our sampled populations or alternatively may be the result of population structure or incomplete population sampling. The latter may be the most likely explanation of the observed null allele frequencies, as four of the five cases of high null allele frequencies occurred in S101 and E405, the least comprehensively sampled populations.

Forty-three of 480 pairs of neutral and immune-linked loci demonstrated significant LD after correction for multiple tests. None of the 144 neutral pairs of loci were in significant LD across all populations, and thus, the observed significant LD values were likely due to population structure. Eleven of 140 pairs of immune-linked loci showed LD, with three MHC class II gene pairs (DRB3 and DRB1; DRB1 and DRA1; DRB4 and DRB1) in LD across the four bobcat populations. DRB and DRA Class II genes are found within 18.3 Mb of each other on chromosome B2 and have related functions. Specifically, DRB1 and DRB3 are separated by 24 kb, DRB1 and DRB4 are separated by 79 kb, and DRB1 and DRA1 are separated by 18.3 Mb. Consequently, because the linkage of these loci may have influenced the results of our analyses, we performed the tests with and without DRB1 and found no apparent change in the results or significance tests (Tables S4 and S9–S10, Fig. S1B).

Seven neutral and three immune-linked loci showed significant deviation from HWE, although none deviated from equilibrium across all four populations (Table S11). Significant heterozygote deficiencies were observed for immune markers including FLA1, DRB1, DRB3, and TLR3 and neutral markers FCA026, FCA090, and FCA086, although not consistently across all four populations (Table S12). Three immune-linked loci (DRB1, DRB3, and TLR3) were observed to be heterozygote deficient in N101 before/during mange, but were not deficient in N101 postmange. Heterozygote excess was not observed for neutral or immune-linked markers for any population.

### Genetic diversity

All loci were polymorphic with 6–15 alleles observed per neutral locus and 3–10 alleles observed per-immune-linked locus. Observed heterozygosity (*H*_O_) ranged from 0.55 to 0.72 for neutral loci, and 0.60 to 0.65 for immune-linked loci (Tables2 and S13). Expected heterozygosity (*H*_E_) ranged from 0.64 to 0.73 for neutral loci and from 0.63 to 0.71 for immune-linked loci. Mean per-locus allelic richness (*A*_R_) ranged from 5.22 to 6.88 for neutral markers, and 4.71 to 6.21 for immune-linked markers (Table2; Table S14). Allelic richness (*χ*^2^* *= 6.93, *P*_neutral_* *= 0.07; *F*_3,13_* *= 0.97, *P*_immune_* *= 0.44), observed heterozygosity (*F*_3,17_* *= 2.60, *P*_neutral_* *= 0.09; *χ*^2^* *= 1.43, *P*_immune_* *= 0.70), and expected heterozygosity (*χ*^2^* *= 7.08, *P*_neutral_* *= 0.07; *F*_3,13_* *= 0.94, *P*_immune_* *= 0.45) did not significantly vary across the four populations for either class of marker. These measures also did not significantly vary for immune-linked loci when DRB1 was excluded (*H*_O_: *F*_3,11_* *= 1.04, *P *=* *0.41; *H*_E_: *F*_3,11_* *= 0.43, *P *=* *0.73; *A*_R_: *χ*^2^* *= 4.59, *P*_neutral_* *= 0.20). However, values for each diversity measure and class of marker were lowest in the most isolated, fragmented eastern population of bobcats (E405).

**Table 2:** Genetic diversity measures for four bobcat populations in SMMNRA. Standard errors are shown in parentheses. Neutral loci measures are shown to the left and immune-linked loci measures are to the right within columns

Population	*H*_O_	*H*_E_	AR	*F*_IS_	*R*
E405	0.55 (0.06)/0.60 (0.05)	0.64 (0.04)/0.63 (0.03)	5.22 (0.57)/4.71 (0.69)	0.14 (0.05)/0.02 (0.04)	0.09 (0.004)
S101	0.65 (0.06)/0.61 (0.05)	0.73 (0.02)/0.70 (0.04)	6.88 (0.73)/6.08 (0.92)	0.09 (0.05)/0.14 (0.05)	0.08 (0.001)
N101-B/DM	0.72 (0.03)/0.64 (0.06)	0.73 (0.03)/0.70 (0.06)	6.84 (0.62)/6.21 (0.80)	0.01 (0.02)/0.08 (0.03)	0.08 (0.001)
N101-PM	0.61 (0.04)/0.65 (0.05)	0.68 (0.03)/0.71 (0.05)	5.93 (0.75)/6.14 (0.76)	0.10 (0.03)/0.03 (0.03)	0.09 (0.003)

SMMNRA, Santa Monica Mountains National Recreation Area.

The disease outbreak resulted in lower genetic diversity measures in the N101 population for neutral loci, though not for immune-linked loci. Neutral loci *H*_O_ were significantly lower (*t*_16_* *= 2.33, *P *=* *0.03) after the mange outbreak (2006–2012) compared with before mange (1996–2001; Table S3). Neutral *H*_E_ and *A*_R_ were also lower post-disease epizootic, although the differences were not significant (Tables2 and S3). A loss of ≥1 rare allele occurred after mange for all neutral loci with the exception of FCA043. With respect to immune-linked loci, *H*_O_, *H*_E,_ and *A*_R_ did not differ significantly before and after the disease outbreak.

The highest inbreeding coefficient (*F*_IS_) values measured using neutral loci were found in E405, the most isolated and fragmented population, and in N101 postmange, the population that suffered a sharp decline as a result of the disease outbreak (Table2). The E405 neutral inbreeding coefficient (*F*_IS_* *= 0.14; SE* *= 0.05) was 14 times greater than N101 before/during mange (*F*_IS_* *= 0.01; SE* *= 0.02), the population with the lowest *F*_IS_. The inbreeding coefficient measured with neutral loci increased 10-fold for the N101 population post-disease (*t*_17_* *= −2.83, *P *=* *0.01) (Table2) compared with before the disease outbreak. The differences in neutral *F*_IS_ across the four populations were significant (*χ*^2^* *= 14.92, *P *=* *0.005). Neutral *F*_IS_ pairwise comparisons between E405 and N101 before/during mange, and N101 before/during mange and postmange, differed significantly (*t*_12_* *= 2.82, *P *=* *0.02 and *t*_17_* *= 2.83, *P *=* *0.01). Neutral loci *F*_IS_ values were also significantly different from zero for E405, S101, and N101 postmange (*P *=* *0.001 for each population).

In contrast, *F*_IS_ measured using immune-linked loci did not vary significantly across the four populations when tested using all seven loci (*F*_3,15_* *= 2.05, *P *=* *0.15) and when DRB1 was excluded (*F*_3,13_* *= 1.15, *P *=* *0.37) (Tables2 and S9). However, the range of *F*_IS_ values for both classes of loci was similar (neutral *F*_IS_* *= 0.01–0.14; immune *F*_IS_* *= 0.02–0.14), and thus, the lack of significant difference across the four populations for immune-linked loci may be due to insufficient power or Type II error. Immune *F*_IS_ values were significantly greater than zero for S101 and N101 before/during mange (*P *=* *0.002 for each population).

Relatedness (Table2) was significantly different across the four populations (*F*_3,131_* *= 3.21, *P *<* *0.001). The significant results reflect the high number of individual pairwise relatedness observations, but the relatedness values are very similar. Thus, the biological significance of the differences is questionable. Within population, relatedness was highest for N101 postmange (*R* = 0.09, SE* *= 0.003) and E405 (*R* = 0.09, SE* *= 0.004) while lowest for S101 (*R* = 0.08, SE* *= 0.001) and N101 before/during mange (*R* = 0.08, SE* *= 0.001). In pairwise comparisons, E405 was significantly higher than S101 (*t*_2694_* *= 3.57, *P *<* *0.001) and N101 before/during mange (*t*_2785_* *= 3.83, *P *<* *0.001). S101 and N101 before/during mange had significantly lower *R* compared with N101 postmange (*t*_2273_* *= −3.99, *P *<* *0.001 and *t*_2333_* *= 4.22, *P *<* *0.001). Relatedness did not significantly differ between E405 and N101 postmange (*t*_4075_* *= −0.6435, *P *=* *0.52) or S101 and N101 before/during mange (*t*_32040_* *= 0.67, *P *=* *0.50).

### Demography

For neutral markers, using the program bottleneck, we detected significant heterozygote excess in the N101 population from 2004 to 2005 (*P *=* *0.007) and 2006 to 2008 (*P *=* *0.02) using both 78% and 90% single-step mutation parameters, evidence that a bottleneck occurred as a result of the disease outbreak (78%: Table3 and 90%: Table S15). We also detected a deviation from the expected L-shaped allele distribution for neutral loci in 2004**–**2005 indicating that a significant shift in allele frequencies occurred as a result of the population decline. We did not detect heterozygote excess for any other population. Using Garza and Williamson's bottleneck test, *M* ratios ranged from 0.67 to 0.89 for neutral loci across all populations and years (Tables3 and S15). We detected a bottleneck in the N101 population during years 2004**–**2005 (*M* = 0.69), 2006**–**2008 (*M* = 0.71), and 2009**–**2012 (*M* = 0.67).

**Table 3:** Results of bottleneck tests using bottleneck and *M*-ratio using 78% single-step mutations

			bottleneck			*M*-ratio *P*		
Population	Year	*N*	TPM	Mode shift	*M*-ratio	*θ *= 0.05	*θ *= 0.1	*θ *= 0.3
N101	1996–1998	34	0.179	No	0.839	0.245	0.282	0.386
	2000–2001	23	0.082	No	0.809	0.145	0.171	0.263
	2002*	22	0.102	No	0.792	0.111	0.130	0.202
	2003*	26	0.326	No	0.810	0.156	0.178	0.259
	2004–2005*	23	**0.002**	**Yes**	**0.690**	**0.011**	**0.012**	**0.021**
	2006–2008	21	**0.007**	No	**0.708**	**0.014**	**0.021**	**0.033**
	2009–2012	31	0.367	No	**0.666**	**0.005**	**0.006**	**0.011**
S101	1996–2001†	30	0.125	No	0.894	0.507	0.545	0.671
	2006–2012†	26	0.064	No	0.788	0.105	0.115	0.183
	2008–2012	85	0.150	No	0.806	0.1500	0.162	0.249
E405	2010–2011	48	0.177	No	0.799	0.165	0.193	0.284

TPM, two-phase model.

Values in bold are significant (*P ≤ *0.05) indicators of a genetic bottleneck. Varying *θ* values correspond with multiple prebottleneck effective population size estimates where *θ *= 0.05 (*N*_e_* *= 25), *θ *= 0.1 (*N*_e_* *= 50), and *θ *= 0.3 (*N*_e_* *= 150).

* Years during which the mange epizootic occurred.

† Malibu Creek State Park subset of S101 population only.

The effective population size, *N*_e_, for bobcats found north of the 101 Freeway (N101) before mange was 47 (95% CI: 25.9–130.7) individuals during 1996–1998 and 17.6 (95% CI: 12.1–27.6) individuals during 2000–2001. The cause of the pre-disease effective population size variation is unknown. However, limited field observations made by NPS do not support that a population decline occurred from 1996 to 2001. Further, the effective population size estimates have overlapping confidence intervals across most years for the N101 population. Therefore, the differences in effective population size pre-disease may be due to stochastic differences in sampling. However, during the final years of the mange outbreak, *N*_e_ was nine (95% CI: 5.6–13.0) individuals (Table1). This decline in the effective population size during the mange outbreak is consistent with field observations of fewer bobcats in N101 as a result of the disease outbreak. For bobcats east of the I-405 (E405), the effective population size was estimated to be 34.4 (95% CI: 21.8–61.2) between years 2010 and 2011. Combining all bobcats captured south of the 101 Freeway (S101) from 1996 to 2012, *N*_e_ was estimated to be 97.7 (95% CI: 71–143.6).

### Population assignments and genetic differentiation

Using the Evanno method to choose the most likely number of population clusters based on a Δ*K* value (Evanno et al. 2005), two (*K *=* *2) clusters had the highest posterior probability assignments for neutral loci in structure (Table S4 and Fig. S2A). However, population clusters resolved at *K *=* *4 corresponded better with our geographic and temporal knowledge of bobcat substructure (Fig.2A) and importantly, Geneland resolved population assignments concordant with the genetic clusters at *K *=* *4 determined in structure (Fig.2D–G). Additionally, the mean LnP(*K*) values generated in structure for each *K* value plateau at *K *=* *9 and do not point to a discrete number of population clusters (Table S4). Evanno et al. (2005) caution that Δ*K* may be used to help identify the correct number of clusters in most situations but that it should not be used exclusively. Partial sampling of individuals in a population has been shown to lead to a lower Δ*K* than the true value, and for a cryptic species such as the bobcat, complete population sampling would not be feasible. Evanno et al. (2005) also suggest that Δ*K* should be considered in conjunction with other information provided by structure such as individual assignment patterns. When we do so, also with consideration of Geneland results, *K *=* *4 is the most biologically sound population clustering.

Geographically, populations from both structure (*K *=* *4) and Geneland included individuals captured east of I-405 (E405), south of the 101 Freeway (S101), and two clusters north of the 101 Freeway (N101) divided temporally. The first N101 population included individuals sampled from 1996 to 2005 (structure) or 1996 to 2001 (Geneland) in the mange-affected region, along with the Moorpark satellite group of individuals (*N* = 10) sampled northwest of the mange-affected region in 2010–2011. The second population north of the 101 Freeway included all animals sampled postepizootic (2006–2012), with the exception of the Moorpark individuals that clustered with N101 before/during mange population. Interestingly, nine samples from GP assign with the E405 population although the park is isolated from this population by the 101 Freeway (Fig.1). When structure was used to examine population turnover in N101 based on survival of individuals over 2-year increments, two clusters (*K *=* *2) were resolved that corresponded with pre- and postbottleneck cluster assignments (Figs2B and S2B, and Table S4). Based on assignment probabilities and posterior probabilities of >50% (Table S16), only one bobcat captured in N101 genetically assigned with the S101 population, while three bobcats captured in S101 genetically assigned to E405.

Both freeways and disease influenced population differentiation (Tables4, S5 and S10). Using neutral markers, all pairwise comparisons of *D*_est_ (Table4) showed genetic differentiation between populations (*D*_est_* *= 0.06–0.12) with a global *D*_est_* *= 0.10. All probability calculations for neutral *D*_est_ were significant at *P *≤* *0.001. The greatest *D*_est_ values were observed between the E405 and N101 populations that are geographically separated by two major freeways and the greatest geographical distance. For the N101 before/during mange and N101 postmange populations *D*_est_* *= 0. 08, a value greater than for those populations separated by a major freeway (Table4).

**Table 4:** Jost's *D*_est_ for nine neutral and seven immune-linked loci, and *P*-values for pairwise comparisons of immune and neutral *D*_est_ estimates for each population pair

	*D*_est_		
Population pairs	Neutral	Immune	*P*
E405*–*S101	0.06	0.09	0.68
E405*–*N101-B/DM	0.11	0.11	0.69
E405*–*N101-PM	0.12	0.17	0.54
S101*–*N101-B/DM	0.05	0.03	0.46
S101*–*N101-PM	0.05	0.04	0.38
N101-B/DM*–*N101-PM	0.08	0.02	**0.03**

All probability calculations for *D*_est_ values are significant. Value in bold represents a significant difference between the two classes of markers at *P ≤ *0.05.

Using immune markers, all pairwise comparisons of *D*_est_ (Table4) also showed genetic differentiation between populations (*D*_est_* *= 0.02–0.17) with a global *D*_est_* *= 0.09. All probability calculations for immune *D*_est_ were significant at *P *≤* *0.002. Using immune markers, overall genetic differentiation between populations was lowest between N101 before/during mange and postmange (*D*_est_* *= 0.02) and highest between E405 and N101 postmange (*D*_est_* *= 0.17).

### Tests of selection

Although all *D*_est_ values were significant, using structure and Geneland, no clear population structure was resolved when tested using immune-linked loci (Figs2C and S1). In pairwise *D*_est_ comparisons of the two classes of markers, a significant difference between neutral and immune-linked loci was found only between N101 before/during mange and postmange (*W *=* *10.5, *P *=* *0.03) (Table4). This finding was consistent when tested without DRB1 (*W *=* *8.5, *P *=* *0.03) (Table S10). Consequently, to evaluate the degree to which fewer immune-linked loci, in comparison with neutral loci, may influence the resolution of population structure, we performed an additional structure analysis with six neutral loci chosen based on their similarities to the immune-linked loci with respect to their number of alleles and allele frequency distributions. We found that less structure was resolved, although more structure than for immune-linked loci when tested using six loci (Fig. S1C). Thus, in conclusion, differences in the number of alleles (Table S17) or allele frequency spectra between the two classes of markers may influence the resolution of population structure. In addition, differential patterns of per-locus selection and drift across the different bobcat populations may also have contributed to the observed absence of structure for immune-linked loci when tested using structure (Fig.2H).

Measures of per-immune-linked locus differentiation for the N101 before/during and post-disease population pair revealed TLR3, TLR4, and FLA1 to each have a *D*_est_* *= 0.00 (Table5). For loci DRA1 and DRB1, *D*_est_ values were lower than overall neutral values (*D*_est_* *= 0.02 and 0.06), while DRB3 and DRB4 had *D*_est_ values similar to the overall neutral value (*D*_est_* *= 0.08 and 0.10). Using probability tests, none of the per-locus *D*_est_ values were significant (Table5) between N101 before/during mange and N101 postmange. Although no outlier loci were detected using lositan, the program authors report that the program is ill-equipped to detect low *F*_ST_ outliers as *F*_ST_ approaches zero (Antao et al. 2008).

**Table 5:** Per-immune locus and overall neutral inbreeding coefficient (*F*_IS_) and genetic differentiation (*D*_est_) values for the N101 populations

		*F*_IS_		
Class	Locus	N101-B/DM	N101-PM	*D*_est_
Neutral	All neutral (SE)	0.01 (0.02)	**0.10** (0.03)	**0.08**
MHC class I	FLA1	**0.10**	0.06	0.00
MHC class II	DRA1	−0.04	0.02	0.02
	DRB1	**0.15**	−0.12	0.06
	DRB3	**0.08**	0.04	0.08
	DRB4	0.06	**0.17**	0.10
TLR	TLR3	**0.16**	−0.06	0.00
	TLR4	0.02	0.02	0.00
Immune	All immune (SE)	**0.08** (0.03)	0.02 (0.03)	0.03

MHC, major histocompatibility complex; TLR, Toll-like receptors.

Standard error (SE) values are in parentheses for composite *F*_IS_ values. Values in bold are significant at *P ≤ *0.05. Significance was calculated using permutation tests.

Inbreeding coefficients showed evidence of both balancing selection and genetic drift acting on immune-linked loci in SMMNRA. The S101 population had the highest inbreeding coefficient at immune-linked loci, greater than for neutral loci and equivalent to the E405 neutral inbreeding coefficient. In contrast, the E405 population had the greatest inbreeding coefficient using neutral loci (Table2), and yet a significantly lower inbreeding coefficient measured using immune-linked loci (*t*_16_* *= −2.26, *P *=* *0.04). Further, despite the 10-fold increase in neutral *F*_IS_ in N101 post-disease, immune-linked loci *F*_IS_ decreased by more than half. The decrease was not significant when comparing N101 before/during mange and postmange (*t*_13_* *= −1.57, *P *=* *0.14), but when N101 before and postmange were compared (Table S3), the difference was significant (*W *=* *13, *P *=* *0.05). Per-locus decreases in *F*_IS_ were also observed for multiple immune-linked loci when comparing N101 before/during mange with postmange (Table5). TLR3, FLA1, DRB1, and DRB3 differed significantly from zero in N101 before/during disease (*P *=* *0.005, 0.03, 0.005, and 0.02), but decreased in N101 postmange and no longer differed significantly from zero. DRB4, in contrast, increased significantly from 0.06 to 0.17.

## Discussion

### Population structure

Urban development is increasingly recognized to have important genetic impacts on wildlife populations (Riley et al. 2006, 2010; Delaney et al. 2010; Munshi-South and Kharchenko 2010; Lee et al. 2012). We evaluated the roles that freeways and disease had on genetic structure and differentiation in urban populations of a highly mobile carnivore. We identified four population groupings that clustered temporally and spatially that corresponded to *K *=* *4 in structure analysis and Geneland. These clusters were comprised of populations separated by freeways or before and after a disease outbreak. Neutral measures of genetic diversity were lowest in the most isolated, fragmented population (E405) and in N101 postmange, a population that suffered a population bottleneck caused by a disease outbreak. These findings indicate the freeways and disease are important contributors to genetic diversity and differentiation in bobcat populations in SMMNRA.

Roads and freeways are a common feature of most landscapes and may impose an important barrier to movement fragmenting animal populations (Forman and Alexander 1998). Within SMMNRA, many secondary roads are present, although they were not observed to contribute to population structure. However, both major freeways in the study area were important barriers to movement and gene flow. The I-405 was a greater barrier to gene flow than the 101 Freeway. Further, as suggested by specific migrant data, dispersal across the I-405 appears unidirectional moving east to west perhaps due to the limited available habitat east of the I-405 (Fig.1). Higher population differentiation across the I-405 may also be the result of fewer potential wildlife crossing points as the I-405 has only four potential points whereas the 101 Freeway has seven. Greater traffic densities on the I-405 compared with the 101 Freeway may also contribute to higher population differentiation across the I-405. The influence of the 101 Freeway on population structure and genetic differentiation is consistent with previous results by Riley et al. (2006) although this study detected 13 migrants whereas we detected only four. However, our dataset includes a greater number of individuals and loci, and so consequently, we potentially have more power to detect true migrants. Further, we sampled approximately twice as many individuals in E405 as in the area directly west of the I-405, so we do not believe our migrant detection for the areas straddling the I-405 to be downwardly biased by our sampling design.

In southern California, a variety of species have been documented to cross freeways through culverts and passageways under freeways (Ng 2004). Riley et al. (2006) found through radio-telemetry observations that only four bobcats crossed the 101 Freeway while using remote cameras, a more recent study also found extremely limited movement across the I-405 (Schoonmaker and Riley 2011). Within SMMNRA, there are no wildlife corridors across freeways, and although underpasses may be used (Ng 2004), our results highlight the need for improved connectivity across increasingly urbanized and fragmented landscapes to prevent population isolation of bobcats and possibly other low-to-moderate abundance species such as mountain lions (*Puma concolor*, Beier et al. 2010), gray foxes (*Urocyon cinereoargenteus*, Temple et al. 2010), grizzly bears (*Ursus arct*os, Gibeau et al. 2002), among others (Ng 2004). The design and implementation of such corridors across large roadways must become an essential feature of developing megacities where the preservation of rare native species at the urban–wildlife interface is a priority.

Our results also indicated that disease can be as strong an influence on population structure as freeways. Genetic differentiation between the N101 before/during mange and the N101 postmange populations was greater than for populations separated by freeways for >60 years. The disease outbreak caused a decline of >50% on the survival rate of bobcats radio-collared by NPS, and uncollared animals were also found dead with the disease (Riley et al. 2010). The population appears to be recovering (S. P. D. Riley, personal communication) and based on the survivor structure analysis, the pattern of genetic turnover suggests that the population was reestablished by a small group of founders that originated from the N101 population. The mode in which new populations are founded will influence the degree of population differentiation (Wade and McCauley 1988). When the number of colonizers is large and from multiple source populations, turnover will have a homogenizing effect decreasing differentiation (Slatkin 1977). If the number of colonizers is small and from only a few source populations, genetic drift is accelerated and turnover can increase population differentiation (Harrison and Hastings 1996; Pannell and Charlesworth 2000). The degree of differentiation between the N101 before/during and post-disease populations and S101 suggests a small number of N101 individuals survived to establish the postmange population, rather than having been repopulated by individuals from nearby populations (e.g., S101 or Moorpark).

### Genetic effects of a population bottleneck

We found evidence that the N101 bobcat population underwent a bottleneck coincident with the notoedric mange outbreak from 2002 to 2005. Although the reliability of various bottleneck tests to detect the occurrence and timing of genetic bottlenecks has been questioned, particularly the *M*-ratio test (Peery et al. 2012), we used multiple approaches and found concordant results across these approaches. Using the test of heterozygosity excess, we detected a genetic bottleneck across two time increments spanning 2004–2008, while using the *M*-ratio test, we detected a genetic bottleneck across three time increments spanning 2004–2012 in the N101 region. Interestingly, *M* ratios are expected to change more slowly after a genetic bottleneck in comparison with levels of heterozygosity excess (Williamson-Natesan 2005), and our findings fit this prediction. Finally, we also detected a decline of the effective population size in the N101 region coincident with the peak of the disease outbreak. Because a bottleneck signature was observed only in N101, the population decline was likely a local event. The strong genetic evidence of a population bottleneck is consistent with field observations made by NPS biologists that have been radio-collaring and tracking bobcats in the area since 1996 (Riley et al. 2007, 2010). These data illustrate the value of coupling genetic and field studies, particularly for secretive wide-ranging carnivores such as bobcats.

With large reductions in effective population size, enhanced genetic changes from more extreme genetic drift include shifts in allele frequencies, loss of rare alleles, and decreased heterozygosity (Nei et al. 1975; Kimura 1985; Dlugosch and Parker 2008). Consistent with the bottleneck in N101, we detected a significant increase in inbreeding and relatedness in the post-disease N101 population based on neutral loci. However, we observed only a slight nonsignificant decrease in allelic richness and observed heterozygosity after the disease epizootic. If population recovery occurs quickly or the bottleneck is small, significant decreases in heterozygosity or allelic richness may not be detected (Nei et al. 1975). Because bobcats are notoriously elusive and difficult to census by direct observation, the rate at which the population recovered is unclear. However, based on capture rates and reproduction observations, the population recovery occurred quickly, after approximately 7 years, or roughly three bobcat generations (Knick et al. 1985), since the beginning of the mange epizootic (S. P. D. Riley NPS, unpublished data).

### Evidence of selection

Strong selection at functional loci may counteract genetic drift and maintain adaptively important genetic variation (Aguilar et al. 2004; Piertney and Oliver 2005; Oliver and Piertney 2012), even in populations that have undergone extreme genetic bottlenecks (Aguilar et al. 2004; Piertney and Oliver 2005; Whittaker et al. 2012). Remarkably, we observed strong effects of genetic drift acting on neutral loci on a short timescale, with rapid genetic turnover in the population occurring during a single generation. Yet, we also observed a significant discrepancy in the degree of genetic differentiation at immune-linked and neutral loci between N101 before/during mange and N101 postmange and a decrease in the immune inbreeding coefficient postmange, evidence of strong balancing selection acting on immune loci during the disease outbreak. These findings emphasize that strong selection can regulate patterns of variation at immune genes and that the intensity of selection acting on immune regions fluctuates over time (Hedrick 2002). Similarly, strong balancing selection over the course of a single generation was observed to maintain MHC allele frequencies in a bottlenecked vole population (*Arvicola terrestris*; Oliver and Piertney 2012), while greater MHC allele frequency variation between successive great reed warbler (*Acrocephalus arundinaceus*) cohorts than expected at random was attributed to strong directional selection favoring specific MHC alleles (Westerdahl et al. 2004). To our knowledge, we are the first to evaluate how genetic drift and selection influence genetic variation at immunologically important loci in an urban free-ranging wildlife population. Further, the majority of immunogenetic studies have focused on MHC, while very few have examined patterns of genetic diversity at TLR loci, particularly in a free-ranging populations.

Among our most striking results was the significant decrease in immune-linked loci *F*_IS_ simultaneous to a significant, 10-fold increase in *F*_IS_ for neutral markers as a result of the disease outbreak. A low inbreeding coefficient at functional genetic regions in comparison with the inbreeding coefficient measured at neutral loci suggests balancing selection (Black et al. 2001; Oliver and Piertney 2012). While changes in *F*_IS_ values for FLA1, DRB1, DRB3, and TLR3 after the disease outbreak imply balancing selection acting on these loci, the inbreeding coefficient significantly increased for DRB4. Balancing selection is unlikely to affect all immune-linked loci, particularly given their varied functional roles in the vertebrate immune system. We know of only one study that has found evidence of balancing selection affecting variation at TLR regions (Ferrer-Admetlla et al. 2008), whereas our results indicated balancing selection on both TLR loci and four of five MHC loci.

Previous studies that have compared the degree of differentiation between neutral markers and immune genes in wildlife populations have reported varying degrees and types of selection to influence divergence in populations (e.g., Bernatchez and Landry 2003; Piertney and Oliver 2005; Cammen et al. 2010; Bollmer et al. 2011; Tschirren et al. 2011). We observed both a lack of genetic differentiation between before/during and post-disease populations for the FLA1 locus, and a decrease in *F*_IS_ for FLA1 locus, DRB1 and DRB3, suggesting MHC class I and class II-mediated immune response may have been important for bobcats infested with notoedric mange. The scabies mite, *Sarcoptes scabiei*, is closely related to notoedric mange mites, and in humans, an immune response dominated by helper T and cytotoxic T cells that interact directly with MHC class I and class II molecules (Huang and Germain 1992) regulates parasite control (Walton 2010). A recent study found evidence that MHC class II *DRB*-mediated immune response to scabies (*S. rupicaprae*) may increase survival of male Alpine chamois (*Rupicapra rupicapra*) during rutting season (Schaschl et al. 2012). We have documented that bobcats with mange experience leukocytosis, and specifically significantly elevated neutrophil, monocyte, and eosinophil counts (Serieys et al. 2013). Although neutrophils have key function in innate immune response, they express MHC class I molecules (Neuman et al. 1992) and are able to stimulate adaptive immune response (Potter and Harding 2001).

Toll-like receptors may similarly have been important to the initiation of bobcat immune response to mange infection. The observed decrease in TLR3 *F*_IS_ and absence of differentiation for TLR3 and TLR4 between N101 before/during mange and N101 postmange suggests TLR involvement during the disease outbreak. TLR signaling activates antigen-presenting cells that support helper and cytotoxic T cell differentiation (Kaisho and Akira 2006). Additionally, monocytes, neutrophils, and eosinophils, each found elevated in bobcats with severe mange (Serieys et al. 2013), express TLR4 (Sabroe et al. 2002; Iwasaki and Medzhitov 2010), and eosinophils also express TLR3 (Månsson et al. 2010). Across SMMNRA, we have tested bobcats for exposure to 10 common feline viral and bacterial pathogens and have not detected unusually high prevalence of any disease with the exception of mange (L. E. K. Serieys, M. Epeldegui, T. C. Armenta, S. VandeWoude, S. Schlottman, S. P.D. Riley, R. K. Wayne and C. Uittenbogaart, unpublished manuscript). Therefore, the observed patterns of selection are likely driven primarily by severe and widespread mange parasitism.

Differentiation in neutral and immune-linked loci is similar and nonsignificant across other populations, suggesting the latter are not experiencing divergent natural selection or under balancing selection in other areas of SMMNRA. We also observe an absence of population structure for immune-linked loci that may reflect the limited power of seven loci, qualitative differences in variation of the neutral and immune markers, as well as differential patterns of drift and selection acting on individual immune-linked loci across the study area. One suggestive finding is that *F*_IS_ for neutral loci is seven times greater than for immune-linked loci in the E405 population, a difference similar to that for the N101 postmange population. Notoedric mange has been observed in E405 since at least 2006 and presently appears to be a primary source of mortality for bobcats in the population (L. E. K. Serieys, unpublished data). Thus, balancing selection may also be an important regulator of genetic variation at immune-linked loci in the E405 population in response to mange parasitism.

### Conservation implications

Our data highlight the importance of increased urbanization, as manifest by increased exposure to anticoagulants and the presence of freeways, on the genetic structure and diversity of animal populations and suggest that efforts to ameliorate these effects are critical to long-term conservation and effective management. Bottlenecks can reduce genetic variation and thereby threaten the long-term viability of wildlife populations (e.g., Roelke et al. 1993; Espeleta et al. 2001; Acevedo-Whitehouse et al. 2006; Culver et al. 2008). We show that balancing selection may act to maintain functionally important genetic variation in the N101 population. However, our results suggest the general effect of notoedric mange is to reduce genetic variation and increase inbreeding which can decrease the capacity of populations to respond to environmental changes, especially at the urban–wildland interface where stress is greater, and can result in inbreeding depression. Construction of wildlife corridors across freeways is one critical response to these genetic changes that may assist in the preservation of genetic diversity across rapidly urbanizing landscapes. Additionally, maintaining habitat with minimal edge is important. For example, in our study area, anticoagulant poisoning occurs primarily in areas that are associated with urban development, likely reflecting the placement of rodenticides outside homes and in outdoor facilities (Serieys et al. unpublished manuscript). Thus, connected habitats with natural buffers where disease or toxicant effects are less extreme and favorable adaptive genetic variation can spread will be critical for the long-term viability of wildlife populations in urban landscapes.

## Data archiving statement

Microsatellite data are available from the Dryad Digital Repository: http://doi.org/10.5061/dryad.7j8q0.

## Supporting Information

**Figure S1.** Results of population structure analyses using immune-linked and neutral loci.

**Figure S2.** The harvested results of structure analyses as plots of mean LnP(*K*) as a function of *K* (with standard deviations) and delta *K* as a function of *K*.

**Table S1.** The number of individuals (*N*) and proportion of each bobcat sample type per population used for genotyping. **Table S2.** Information for immune-linked microsatellite loci and primers developed for this study. **Table S3.** Genetic diversity measures for the N101 population stratified by mange status: before, during, and after the mange epizootic. **Table S4.** The harvested results of structure analyses. **Table S5.***F*_ST_ values for each population pair calculated using nine neutral loci and seven immune-linked loci. **Table S6.** The proportion of missing data for each locus per population. **Table S7.** Neutral locus null allele frequency estimates per population using four methods implemented in Micro-checker. **Table S8.** Immune-linked locus null allele frequency estimates per population using four methods implemented in Micro-checker. **Table S9.** Genetic diversity measures calculated using immune-linked loci for each bobcat population. **Table S10.** Jost's *D*_est_ for each population pair for nine neutral and six immune-linked loci (DRB1 excluded), and *P*-values for pairwise comparisons of immune and neutral *D*_est_ estimates of genetic differentiation for each population pair. **Table S11.** Per locus Hardy–Weinberg probability values for each population. **Table S12.** Per locus heterozygosity excess and deficiency probability values for each population and across all populations. **Table S13.** Per locus observed and expected heterozygosities per population. Expected heterozygosities are in parentheses. **Table S14.** Allelic richness per locus per bobcat population. **Table S15.** Results of bottleneck tests using bottleneck and *M*-ratio using 90% single-step mutations. **Table S16.**structure cluster assignment and posterior probability of correct genetic population assignment of potential migrants in SMMNRA. **Table S17.** The number of alleles per locus per population.
