# Supplementary figures and images for: Disease and freeways drive genetic change in urban bobcat populations
Source: Evol Appl. 2014 Dec 2;8(1):75–92. doi: 10.1111/eva.12226 (PMC4310583; doi:10.1111/eva.12226)

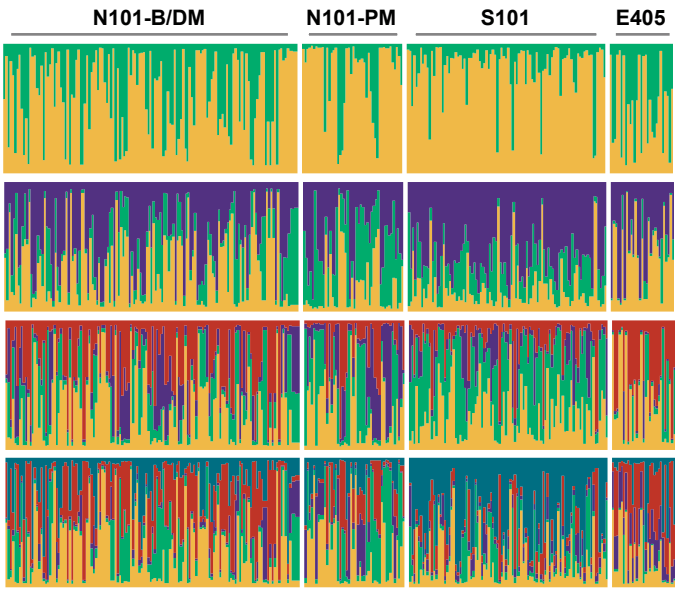

**A**  $K = 2-5$  using seven immune loci.

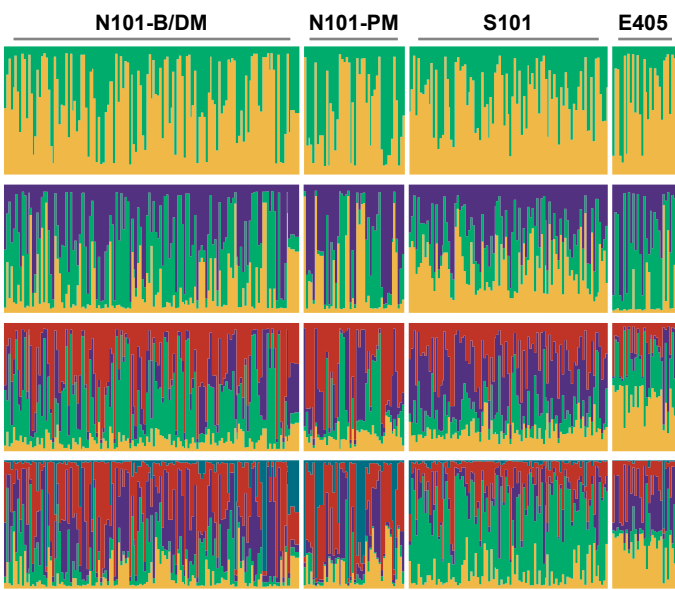

**B**  $K = 2-5$  using six immune loci (DRB1 excluded).

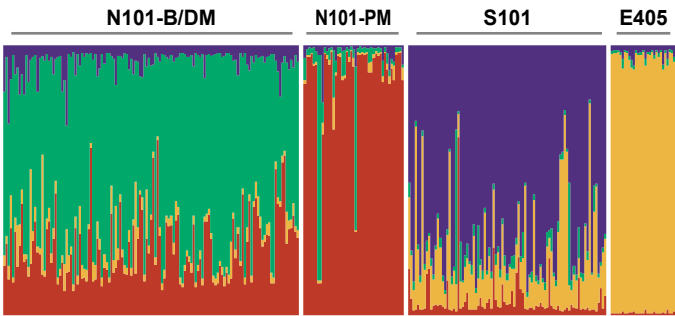

**C**  $K = 4$  using six neutral loci.

Supplement: Supplementary file 1 [file eva0008-0075-sd1.pdf]
