# Supplementary material for: Disease and freeways drive genetic change in urban bobcat populations
Source: Evol Appl. 2014 Dec 2;8(1):75–92. doi: 10.1111/eva.12226 (PMC4310583; doi:10.1111/eva.12226)
Supplement: Supplementary file 2 [file eva0008-0075-sd2.pdf]

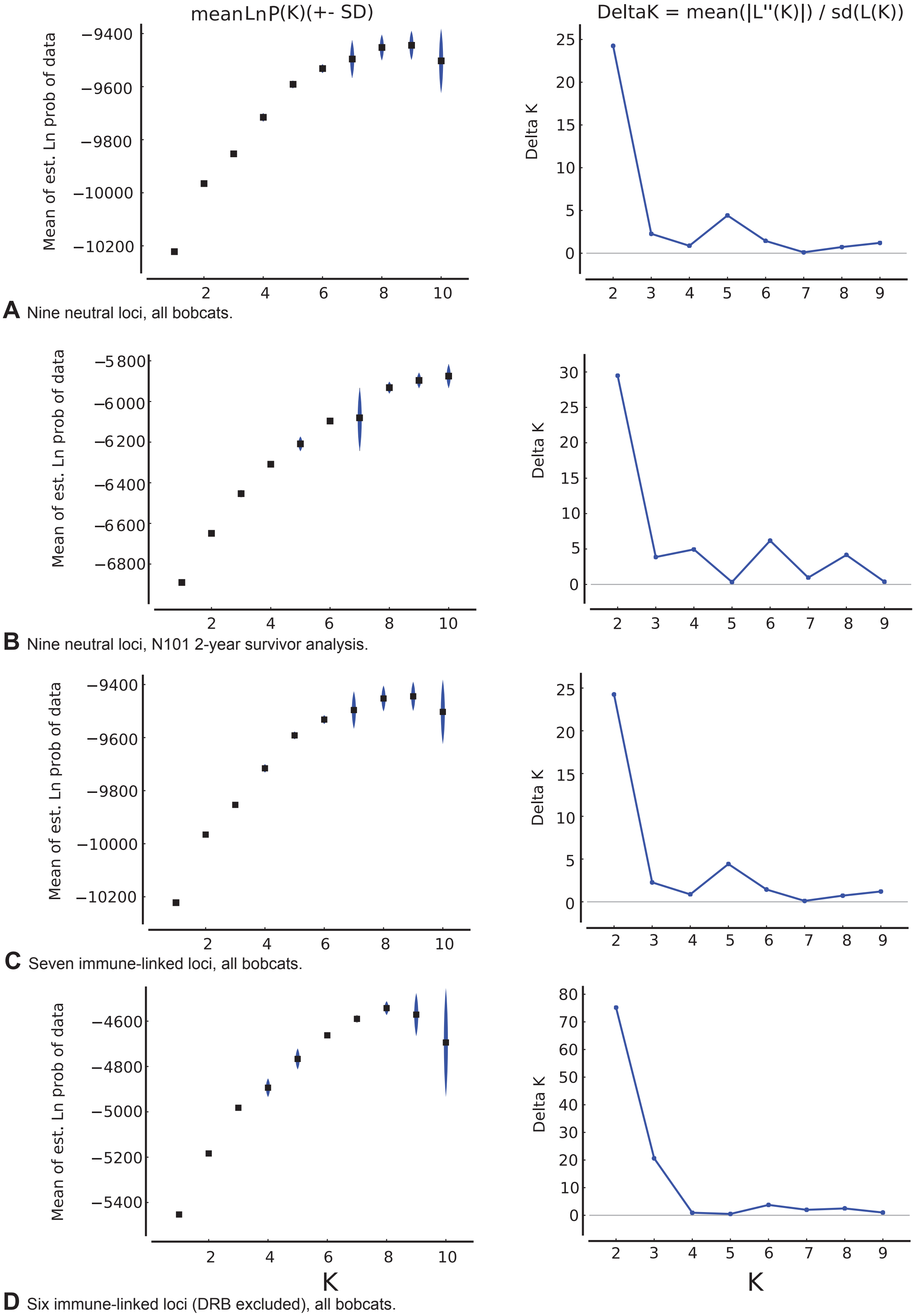
meanLnP(K)(+- SD)
DeltaK = mean(|L''(K)|) / sd(L(K))
Mean of est. Ln prob of data
Delta K
A Nine neutral loci, all bobcats.
B Nine neutral loci, N101 2-year survivor analysis.
C Seven immune-linked loci, all bobcats.
K
K
D Six immune-linked loci (DRB excluded), all bobcats.
