# Supplementary material for: Disease and freeways drive genetic change in urban bobcat populations
Source: Evol Appl. 2014 Dec 2;8(1):75–92. doi: 10.1111/eva.12226 (PMC4310583; doi:10.1111/eva.12226)
Supplement: Supplementary file 3 [file eva0008-0075-sd3.docx]

Table S1. The number of individuals (N) and proportion of each bobcat sample type per population used for genotyping. The values to the left of the backslash represent proportions of each sample type for genotyping using neutral loci, while the values to the right are for immune-linked loci.

| Population | N | Antemortem tissue | Postmortem tissue | Buccal swab | Scat |
| --- | --- | --- | --- | --- | --- |
| E405 | 49 / 29 | 0.56 / 0.91 | 0.09 / 0.03 | 0.00 / 0.00 | 0.35 / 0.00 |
| S101 | 124 / 91 | 0.59 / 0.82 | 0.22 / 0.12 | 0.05 / 0.06 | 0.14 / 0.00 |
| N101-B/DM | 141 / 135 | 0.94 / 0.98 | 0.04 / 0.01 | 0.01 / 0.01 | 0.01 / 0.00 |
| N101-PM | 51 / 44 | 0.72 / 0.84 | 0.06 / 0.00 | 0.14 / 0.16 | 0.08 / 0.00 |

Table S2. Information for immune-linked microsatellite loci and primers developed for this study.

| Locus | Primer (5′–3′) | Length (kb) | Repeat | Relation to target region |
| --- | --- | --- | --- | --- |
| FLA1 | F: ACACACTGAGCACCAAGCAC | 189 | CA | 20 kb downstream of FLA1 |
|  | R: CCCTGCTCACACTCTGTCTG |  |  |  |
| TLR3 | F: CCCCTCCAGTTCTGCAATAA | 267 | TG | 8 kb downstream from TLR3 |
|  | R: GCGAGACTGTAGGCAGTTCC |  |  |  |
| TLR4 | F: GCTTCTCCCTAAATGCTGCC | 251 | TGGA | Intron of TLR4 |
|  | R: ACCTCAATGGACTGCCCTC |  |  |  |
| DRA1 | F: CCCGTGCCTGTTATCAACTT | 216 | GA | 2 kb upstream from DRA1 |
|  | R: GGGTATGATGCCTTCTCCAA |  |  |  |
| DRB1 | F: GCCCTGATGAGGTCAGCC | 287 | TTTA | 5 kb upstream from DRB1 |
|  | R: GATAGAGTCCCAGGTCGGG |  |  |  |
| DRB3 | F: TCTCACGTTTTGTGGGTGTG | 248 | CT | 14 kb upstream from DRB3 |
|  | R: TGAATCCTTCTTGCGGAACT |  |  |  |
| DRB4 | F: ATGGCTCCCAAGGCAAAGG | 273 | CA | Intron of DRB4 |
|  | R: CAAGAGTTGCATGCCCTACC |  |  |  |

Table S3. Genetic diversity measures for the N101 population stratified by mange status: before, during, and after the mange epizootic. Values calculated with nine neutral loci are on the left, and seven immune-linked loci are to the right of the backslash.

| Mange status | Years | N | *H_O_* | *H_E_* | *AR* | F*_IS_* |
| --- | --- | --- | --- | --- | --- | --- |
| Before | 1996-2001 | 56 / 53 | 0.73 / 0.65 | 0.73 / 0.71 | 7.02 / 6.63 | -0.01 / 0.08 |
| During | 2002-2005 | 71 / 70 | 0.71 / 0.62 | 0.73 / 0.68 | 6.19 / 6.32 | 0.02 / 0.09 |
| After | 2006-2012 | 51 / 46 | 0.61 / 0.65 | 0.68 / 0.71 | 5.99 / 6.14 | 0.10 / 0.03 |

Table S4. The harvested results of STRUCTURE analyses. Using the Evanno method, K = 2 (in bold) was the most optimal number of clusters for each analysis shown.

| Analysis | K | Reps | Mean LnP(K) | Stdev LnP(K) | Ln'(K) | \|Ln''(K)\| | Delta K |
| --- | --- | --- | --- | --- | --- | --- | --- |
| Nine neutral loci, all bobcats | 1 | 10 | -10222.14 | 0.2 | – | – | – |
|  | **2** | **10** | **-9965.61** | **5.96** | **256.53** | **144.44** | **24.25** |
|  | 3 | 10 | -9853.52 | 11.27 | 112.09 | 25.63 | 2.27 |
|  | 4 | 10 | -9715.8 | 15.69 | 137.72 | 13.65 | 0.87 |
|  | 5 | 10 | -9591.73 | 14.58 | 124.07 | 64.45 | 4.42 |
|  | 6 | 10 | -9532.11 | 16.47 | 59.62 | 23.71 | 1.44 |
|  | 7 | 10 | -9496.2 | 70.86 | 35.91 | 7.66 | 0.11 |
|  | 8 | 10 | -9452.63 | 48.97 | 43.57 | 35.39 | 0.72 |
|  | 9 | 10 | -9444.45 | 55.24 | 8.18 | 66.86 | 1.21 |
|  | 10 | 10 | -9503.13 | 121.77 | -58.68 | – | – |
| Nine neutral loci, N101 2-year survivor | 1 | 10 | -6890.65 | 0.21 | – | – | – |
|  | **2** | **10** | **-6648.92** | **1.57** | **241.73** | **46.38** | **29.48** |
|  | 3 | 10 | -6453.57 | 13.07 | 195.35 | 50.54 | 3.87 |
|  | 4 | 10 | -6308.76 | 9.02 | 144.81 | 44.59 | 4.95 |
|  | 5 | 10 | -6208.54 | 36.55 | 100.22 | 12.16 | 0.33 |
|  | 6 | 10 | -6096.16 | 16.9 | 112.38 | 104.47 | 6.18 |
|  | 7 | 10 | -6088.25 | 155.34 | 7.91 | 148.55 | 0.96 |
|  | 8 | 10 | -5931.79 | 29.09 | 156.46 | 120.96 | 4.16 |
|  | 9 | 10 | -5896.29 | 38.2 | 35.5 | 14.29 | 0.37 |
|  | 10 | 10 | -5875.08 | 58.58 | 21.21 | – | – |
| Seven immune-linked loci, all bobcats | 1 | 10 | -10222.14 | 0.2 | – | – | – |
|  | **2** | **10** | **-9965.61** | **5.96** | **256.53** | **144.44** | **24.25** |
|  | 3 | 10 | -9853.52 | 11.27 | 112.09 | 25.63 | 2.27 |
|  | 4 | 10 | -9715.8 | 15.69 | 137.72 | 13.65 | 0.87 |
|  | 5 | 10 | -9591.73 | 14.58 | 124.07 | 64.45 | 4.42 |
|  | 6 | 10 | -9532.11 | 16.47 | 59.62 | 23.71 | 1.44 |
|  | 7 | 10 | -9496.2 | 70.86 | 35.91 | 7.66 | 0.11 |
|  | 8 | 10 | -9452.63 | 48.97 | 43.57 | 35.39 | 0.72 |
|  | 9 | 10 | -9444.45 | 55.24 | 8.18 | 66.86 | 1.21 |
|  | 10 | 10 | -9503.13 | 121.77 | -58.68 | – | – |
| Six immune-linked loci (DRB1 excluded), all bobcats | 1 | 10 | -5452.85 | 0.14 | – | – | – |
|  | **­­** | **10** | **-5183.59** | **0.9** | **269.26** | **67.67** | **75.19** |
|  | 3 | 10 | -4982 | 5.48 | 201.59 | 113.03 | 20.62 |
|  | 4 | 10 | -4893.44 | 41.27 | 88.56 | 38.32 | 0.93 |
|  | 5 | 10 | -4766.56 | 46.5 | 126.88 | 22.69 | 0.49 |
|  | 6 | 10 | -4662.37 | 8.43 | 104.19 | 31.63 | 3.75 |
|  | 7 | 10 | -4589.81 | 12.72 | 72.56 | 25.2 | 1.98 |
|  | 8 | 10 | -4542.45 | 30.7 | 47.36 | 76.06 | 2.48 |
|  | 9 | 10 | -4571.15 | 95.4 | -28.7 | 94.26 | 0.99 |
|  | 10 | 10 | -4694.11 | 240.33 | -122.96 | – | – |

Table S5. *F*_ST_ values for each population pair calculated using nine neutral loci and seven immune-linked loci.

| Population pairs | F*_ST_* | |
| --- | --- | --- |
|  | Neutral | Immune |
| E405 *–* S101 | 0.065 | 0.062 |
| E405 *–* N101-B/DM | 0.067 | 0.068 |
| E405 *–* N101-PM | 0.102 | 0.094 |
| S101 *–* N101-B/DM | 0.035 | 0.017 |
| S101 *–* N101-PM | 0.035 | 0.026 |
| N101-B/DM *–* N101-PM | 0.048 | 0.010 |

Table S6. The proportion of missing data for each locus per population. The sample type and number of samples for which data are missing are specified in parentheses.

| Locus | E405 | S101 | N101-B/DM | N101-PM |
| --- | --- | --- | --- | --- |
| FCA008 | 0.00 | 0.02 (N = 3, scat) | 0.00 | 0.00 |
| FCA023 | 0.00 | 0.01 (N = 1, scat) | 0.00 | 0.00 |
| FCA026 | 0.00 | 0.00 | 0.00 | 0.00 |
| FCA043 | 0.00 | 0.01 (N = 1, antemortem tissue) | 0.00 | 0.00 |
| FCA045 | 0.00 | 0.00 | 0.00 | 0.00 |
| FCA077 | 0.00 | 0.01 (N = 1, postmortem tissue) | 0.00 | 0.00 |
| FCA090 | 0.02 (N = 1, scat) | 0.00 | 0.00 | 0.02 (N = 1, scat) |
| FCA096 | 0.00 | 0.02 (N = 2, buccal swab; N = 1, scat) | 0.00 | 0.00 |
| FCA132 | 0.00 | 0.01 (N = 1, scat) | 0.00 | 0.02 (N = 1, scat) |
| FLA1 | 0.00 | 0.01 (N = 1, post-mortem tissue) | 0.02 (N = 3, antemortem tissue) | 0.00 |
| DRA1 | 0.00 | 0.00 | 0.00 | 0.00 |
| DRB1 | 0.00 | 0.00 | 0.01 (N = 1, antemortem tissue) | 0.00 |
| DRB3 | 0.00 | 0.01 (N = 1, post-mortem tissue) | 0.00 | 0.00 |
| DRB4 | 0.00 | 0.02 (N = 1, postmortem tissue; N = 1, antemortem tissue) | 0.00 | 0.00 |
| TLR3 | 0.00 | 0.00 | 0.01 (N = 1, antemortem tissue) | 0.00 |
| TLR4 | 0.00 | 0.00 | 0.00 | 0.00 |

Table S7. Neutral locus null allele frequency estimates per population using four methods implemented in Micro-checker.

| Population | Locus | Evidence of null alleles? | Oosterhout | Chakraborty | Brookfield 1 | Brookfield 2 |
| --- | --- | --- | --- | --- | --- | --- |
| E405 | FCA008 | no | -0.02 | -0.02 | -0.02 | 0.00 |
|  | FCA023 | no | 0.04 | 0.03 | 0.02 | 0.02 |
|  | FCA026 | no | 0.02 | 0.02 | 0.02 | 0.02 |
|  | FCA043 | no | 0.09 | 0.11 | 0.07 | 0.07 |
|  | FCA045 | yes | 0.12 | 0.14 | 0.08 | 0.08 |
|  | FCA077 | no | 0.08 | 0.10 | 0.07 | 0.07 |
|  | FCA090 | yes | 0.17 | 0.24 | 0.11 | 0.18 |
|  | FCA096 | yes | 0.16 | 0.21 | 0.13 | 0.13 |
|  | FCA132 | no | -0.02 | -0.02 | -0.01 | 0.00 |
| S101 | FCA008 | no | -0.01 | -0.01 | 0.00 | 0.07 |
|  | FCA023 | no | 0.01 | 0.01 | 0.01 | 0.05 |
|  | FCA026 | no | 0.00 | 0.00 | 0.00 | 0.00 |
|  | FCA043 | no | 0.01 | 0.02 | 0.01 | 0.05 |
|  | FCA045 | yes | 0.23 | 0.33 | 0.19 | 0.19 |
|  | FCA077 | no | 0.04 | 0.05 | 0.04 | 0.07 |
|  | FCA090 | no | 0.04 | 0.03 | 0.03 | 0.03 |
|  | FCA096 | yes | 0.07 | 0.07 | 0.05 | 0.11 |
|  | FCA132 | no | 0.00 | 0.00 | 0.00 | 0.06 |
| N101-B/DM | FCA008 | no | 0.00 | 0.00 | 0.00 | 0.00 |
|  | FCA023 | no | -0.05 | -0.05 | -0.04 | 0.00 |
|  | FCA026 | yes | 0.07 | 0.07 | 0.06 | 0.06 |
|  | FCA043 | no | 0.03 | 0.03 | 0.03 | 0.03 |
|  | FCA045 | no | 0.00 | 0.00 | 0.00 | 0.00 |
|  | FCA077 | no | -0.03 | -0.03 | -0.02 | 0.00 |
|  | FCA090 | no | 0.02 | 0.03 | 0.02 | 0.02 |
|  | FCA096 | no | 0.03 | 0.02 | 0.02 | 0.02 |
|  | FCA132 | no | -0.02 | -0.01 | -0.01 | 0.00 |
| N101-PM | FCA008 | no | 0.03 | 0.03 | 0.02 | 0.02 |
|  | FCA023 | no | 0.05 | 0.05 | 0.04 | 0.04 |
|  | FCA026 | no | 0.03 | 0.04 | 0.03 | 0.03 |
|  | FCA043 | no | 0.04 | 0.04 | 0.03 | 0.03 |
|  | FCA045 | yes | 0.08 | 0.10 | 0.07 | 0.07 |
|  | FCA077 | no | -0.03 | -0.03 | -0.02 | 0.00 |
|  | FCA090 | yes | 0.13 | 0.15 | 0.09 | 0.19 |
|  | FCA096 | no | 0.05 | 0.05 | 0.04 | 0.04 |
|  | FCA132 | no | 0.07 | 0.06 | 0.05 | 0.11 |

Table S8. Immune-linked locus null allele frequency estimates per population using four methods implemented in Micro-checker.

| Population | Locus | Evidence of null alleles? | Oosterhout | Chakraborty | Brookfield 1 | Brookfield 2 |
| --- | --- | --- | --- | --- | --- | --- |
| E405 | FLA1 | no | 0.11 | 0.11 | 0.07 | 0.07 |
|  | DRB3 | no | 0.00 | 0.02 | 0.02 | 0.02 |
|  | DRB1 | no | -0.05 | -0.05 | -0.04 | 0.00 |
|  | DRA1 | no | -0.01 | -0.01 | -0.01 | 0.00 |
|  | TLR3 | no | -0.01 | 0.00 | 0.00 | 0.00 |
|  | TLR4 | no | 0.04 | 0.04 | 0.03 | 0.03 |
|  | DRB4 | no | -0.05 | -0.04 | -0.04 | 0.00 |
| S101 | FLA1 | yes | 0.19 | 0.23 | 0.16 | 0.18 |
|  | DRB3 | yes | 0.09 | 0.10 | 0.08 | 0.11 |
|  | DRB1 | no | 0.04 | 0.05 | 0.04 | 0.04 |
|  | DRA1 | no | -0.02 | -0.02 | -0.01 | 0.00 |
|  | TLR3 | no | 0.04 | 0.04 | 0.03 | 0.03 |
|  | TLR4 | yes | 0.11 | 0.13 | 0.07 | 0.07 |
|  | DRB4 | no | 0.02 | 0.02 | 0.02 | 0.08 |
| N101-B/DM | FLA1 | no | 0.05 | 0.05 | 0.04 | 0.12 |
|  | DRB3 | yes | 0.04 | 0.04 | 0.03 | 0.03 |
|  | DRB1 | yes | 0.08 | 0.08 | 0.06 | 0.09 |
|  | DRA1 | no | -0.03 | -0.03 | -0.02 | 0.00 |
|  | TLR3 | yes | 0.07 | 0.08 | 0.06 | 0.09 |
|  | TLR4 | no | 0.01 | 0.01 | 0.00 | 0.00 |
|  | DRB4 | no | 0.03 | 0.03 | 0.02 | 0.02 |
| N101-PM | FLA1 | no | 0.03 | 0.03 | 0.03 | 0.03 |
|  | DRB3 | no | 0.02 | 0.02 | 0.02 | 0.08 |
|  | DRB1 | no | -0.09 | -0.07 | -0.06 | 0.00 |
|  | DRA1 | no | 0.02 | 0.01 | 0.01 | 0.01 |
|  | TLR3 | no | -0.04 | -0.03 | -0.02 | 0.00 |
|  | TLR4 | no | 0.01 | 0.02 | 0.01 | 0.01 |
|  | DRB4 | yes | 0.09 | 0.10 | 0.08 | 0.08 |

Table S9. Genetic diversity measures calculated using immune-linked loci for each bobcat population. The values for the total seven immune-linked loci are to the left of the backslash, and the values calculated using six loci (DRB1 excluded) are to the right.

| Statistic | E405 | S101 | N101-B/DM | N101-PM |
| --- | --- | --- | --- | --- |
| Allelic richness | 4.57 / 4.17 | 6.25 / 6.02 | 6.21 / 5.92 | 6.23 / 5.90 |
| F*_IS_* | 0.02 / 0.04 | 0.14 / 0.15 | 0.08 / 0.07 | 0.03 / 0.05 |
| Observed heterozygosity | 0.60 / 0.58 | 0.61 / 0.61 | 0.64 / 0.65 | 0.65 / 0.65 |
| Expected heterozygosity | 0.63 / 0.62 | 0.70 / 0.71 | 0.70 / 0.70 | 0.71 / 0.71 |

Table S10. Jost’s D_est_ for each population pair for nine neutral and six immune-linked loci (DRB1 excluded), and *P*-values for pairwise comparisons of immune and neutral D_est_ estimates of genetic differentiation for each population pair. Value in bold is significant at *P* *≤* 0.05.

| Population pairs | D_est_ | | *P* |
| --- | --- | --- | --- |
|  | Neutral | Immune |  |
| E405 *–* S101 | 0.06 | 0.08 | 0.86 |
| E405 *–* N101-B/DM | 0.11 | 0.09 | 0.69 |
| E405 *–* N101-PM | 0.12 | 0.15 | 0.61 |
| S101 *–* N101-B/DM | 0.05 | 0.02 | 0.38 |
| S101 *–* N101-PM | 0.05 | 0.03 | 0.36 |
| N101-B/DM *–* N101-PM | 0.08 | 0.01 | **0.03** |

Table S11. Per locus Hardy-Weinberg probability values for each population.

| Locus type | Locus | E405 | S101 | N101-B/DM | N101-PM | Across all populations |
| --- | --- | --- | --- | --- | --- | --- |
| Neutral | FCA008 | 0.735 | 0.126 | 0.609 | 0.225 | 0.004 |
|  | FCA023 | 0.084 | 0.297 | 0.401 | 0.023 | 0.001 |
|  | FCA026 | 0.938 | 0.000 | 0.000 | 0.124 | 0.000 |
|  | FCA043 | 0.316 | 0.516 | 0.375 | 0.091 | 0.000 |
|  | FCA045 | 0.546 | 0.000 | 0.401 | 0.439 | 0.000 |
|  | FCA077 | 0.030 | 0.485 | 0.001 | 0.358 | 0.000 |
|  | FCA090 | 0.000 | 0.581 | 0.024 | 0.031 | 0.013 |
|  | FCA096 | 0.076 | 0.151 | 0.046 | 0.163 | 0.000 |
|  | FCA132 | 0.134 | 0.039 | 0.099 | 0.009 | 0.000 |
| Immune | FLA1 | 0.049 | 0.000 | 0.611 | 0.198 | 0.000 |
|  | DRA1 | 0.162 | 0.411 | 0.501 | 0.347 | 0.000 |
|  | DRB1 | 0.874 | 0.151 | 0.026 | 0.254 | 0.017 |
|  | DRB3 | 0.051 | 0.058 | 0.066 | 0.357 | 0.137 |
|  | DRB4 | 0.396 | 0.043 | 0.055 | 0.001 | 0.062 |
|  | TLR3 | 0.865 | 0.753 | 0.013 | 0.306 | 0.000 |
|  | TLR4 | 0.687 | 0.033 | 0.455 | 0.669 | 0.081 |

Table S12. Per locus heterozygosity excess and deficiency probability values for each population and across all populations. Values in bold are significant at *P* *≤* 0.05 after correction for 32 test statistical tests per population (α = 0.002). N101-B/DM: N101 before/during mange; N101-PM: N101 post-mange.

| Locus type |  | **All** | | **E405** | | **S101** | | **N101-B/DM** | | **N101-PM** | |
| --- | --- | --- | --- | --- | --- | --- | --- | --- | --- | --- | --- |
|  | Locus | Excess | Deficiency | Excess | Deficiency | Excess | Deficiency | Excess | Deficiency | Excess | Deficiency |
| Neutral | FCA008 | 0.297 | 0.703 | 0.371 | 0.654 | 0.387 | 0.615 | 0.851 | 0.158 | 0.012 | 0.988 |
|  | FCA023 | 0.583 | 0.417 | 0.847 | 0.155 | 0.638 | 0.362 | 0.987 | 0.013 | 0.036 | 0.964 |
|  | FCA026 | 1.000 | **0.000** | 0.809 | 0.192 | 0.259 | 0.741 | 0.847 | 0.156 | 0.995 | 0.005 |
|  | FCA043 | 0.912 | 0.088 | 0.97 | 0.031 | 0.879 | 0.122 | 0.651 | 0.353 | 0.372 | 0.628 |
|  | FCA045 | 1.000 | **0.000** | 0.992 | 0.01 | 1.000 | **0.000** | 0.999 | **0.001** | 0.059 | 0.941 |
|  | FCA077 | 0.926 | 0.074 | 0.968 | 0.033 | 0.965 | 0.035 | 0.318 | 0.688 | 0.018 | 0.982 |
|  | FCA090 | 1.000 | **0.000** | 1.000 | **0.000** | 0.875 | 0.126 | 0.961 | 0.04 | 0.989 | 0.011 |
|  | FCA096 | 1.000 | **0.000** | 1.000 | **0.000** | 0.973 | 0.027 | 0.841 | 0.167 | 0.13 | 0.871 |
|  | FCA132 | 0.911 | 0.089 | 0.855 | 0.145 | 0.736 | 0.264 | 0.733 | 0.268 | 0.434 | 0.566 |
| Immune | FLA1 | 1.000 | **0.000** | 0.963 | 0.039 | 1.000 | **0.001** | 0.978 | 0.022 | 0.988 | 0.012 |
|  | DRA1 | 0.950 | 0.05 | 0.593 | 0.554 | 0.536 | 0.465 | 0.179 | 0.821 | 0.469 | 0.536 |
|  | DRB1 | 0.999 | **0.001** | 0.351 | 0.676 | 0.988 | 0.013 | 0.998 | **0.002** | 0.112 | 0.907 |
|  | DRB3 | 1.000 | **0.000** | 0.995 | 0.005 | 0.997 | 0.003 | 0.999 | **0.001** | 0.816 | 0.184 |
|  | DRB4 | 0.997 | 0.004 | 0.431 | 0.605 | 0.764 | 0.237 | 0.813 | 0.187 | 0.988 | 0.012 |
|  | TLR3 | 0.993 | 0.007 | 0.55 | 0.464 | 0.867 | 0.135 | 0.999 | **0.001** | 0.365 | 0.639 |
|  | TLR4 | 0.939 | 0.061 | 0.806 | 0.376 | 0.993 | 0.023 | 0.738 | 0.294 | 0.652 | 0.448 |

Table S13. Per locus observed and expected heterozygosities per population. Expected heterozygosities are in parentheses.

| Locus type | Locus | E405 | S101 | N101-B/DM | N101-PM |
| --- | --- | --- | --- | --- | --- |
| Neutral | FCA008 | 0.70 (0.67) | 0.76 (0.78) | 0.75 (0.75) | 0.62 (0.66) |
|  | FCA023 | 0.58 (0.64) | 0.69 (0.74) | 0.88 (0.78) | 0.62 (0.69) |
|  | FCA026 | 0.82 (0.85) | 0.89 (0.85) | 0.70 (0.80) | 0.75 (0.81) |
|  | FCA043 | 0.52 (0.62) | 0.65 (0.67) | 0.69 (0.74) | 0.64 (0.69) |
|  | FCA045 | 0.40 (0.53) | 0.29 (0.61) | 0.76 (0.76) | 0.52 (0.64) |
|  | FCA077 | 0.52 (0.63) | 0.64 (0.76) | 0.81 (0.77) | 0.77 (0.74) |
|  | FCA090 | 0.29 (0.45) | 0.60 (0.68) | 0.52 (0.55) | 0.39 (0.54) |
|  | FCA096 | 0.40 (0.63) | 0.56 (0.67) | 0.64 (0.68) | 0.62 (0.69) |
|  | FCA132 | 0.74 (0.72) | 0.80 (0.78) | 0.77 (0.73) | 0.57 (0.65) |
| Immune | FLA1 | 0.45 (0.61) | 0.47 (0.74) | 0.67 (0.75) | 0.65 (0.75) |
|  | DRA1 | 0.65 (0.65) | 0.73 (0.72) | 0.79 (0.76) | 0.78 (0.74) |
|  | DRB1 | 0.74 (0.71) | 0.60 (0.66) | 0.61 (0.72) | 0.67 (0.72) |
|  | DRB3 | 0.58 (0.65) | 0.67 (0.83) | 0.78 (0.85) | 0.76 (0.85) |
|  | DRB4 | 0.65 (0.63) | 0.76 (0.80) | 0.74 (0.80) | 0.72 (0.80) |
|  | TLR3 | 0.71 (0.70) | 0.61 (0.66) | 0.53 (0.63) | 0.60 (0.67) |
|  | TLR4 | 0.42 (0.47) | 0.39 (0.49) | 0.38 (0.39) | 0.41 (0.44) |

Table S14. Allelic richness per locus per bobcat population. N101-B/DM: N101 before/during mange; N101-PM: N101 post-mange.

| Locus type | Locus | E405 | S101 | N101-B/DM | N101-PM |
| --- | --- | --- | --- | --- | --- |
| Neutral | FCA008 | 5.00 | 6.52 | 4.90 | 5.97 |
|  | FCA023 | 4.00 | 4.91 | 4.90 | 5.86 |
|  | FCA026 | 9.00 | 12.11 | 10.70 | 12.45 |
|  | FCA043 | 4.00 | 4.94 | 4.99 | 4.99 |
|  | FCA045 | 3.00 | 6.84 | 6.00 | 7.30 |
|  | FCA077 | 5.96 | 6.18 | 6.00 | 6.57 |
|  | FCA090 | 5.00 | 5.85 | 4.92 | 5.06 |
|  | FCA096 | 5.00 | 6.75 | 6.00 | 6.86 |
|  | FCA132 | 5.98 | 7.84 | 4.92 | 6.51 |
| Immune | FLA1 | 4.00 | 6.52 | 6.37 | 7.63 |
|  | DRB3 | 3.00 | 4.00 | 4.00 | 4.00 |
|  | DRB1 | 7.00 | 7.67 | 7.99 | 8.23 |
|  | DRA1 | 4.00 | 9.15 | 8.02 | 7.17 |
|  | TLR3 | 6.00 | 7.22 | 6.67 | 5.99 |
|  | TLR4 | 2.00 | 2.00 | 2.63 | 2.99 |
|  | DRB4 | 6.00 | 7.21 | 7.82 | 7.62 |

Table S15. Results of bottleneck tests using BOTTLENECK and *M*-ratio using 90% single-step mutations. Values in bold are significant (*P* *≤* 0.05) indicators of a genetic bottleneck. Varying *θ* values correspond with multiple pre-bottleneck effective population size estimates where *θ* = 0.05 (*N_e_* = 25), *θ* = 0.1 (*N_e_* = 50), and *θ* = 0.3 (*N_e_* = 150).

|  | Year | N | BOTTLENECK | | *M*-ratio | *M*-ratio *P* | | |
| --- | --- | --- | --- | --- | --- | --- | --- | --- |
| Population |  |  | TPM‡ | Mode shift |  | *θ* = 0.05 | *θ* = 0.1 | *θ* = 0.3 |
| N101 | 1996-1998 | 34 | 0.455 | no | 0.839 | 0.04 | 0.051 | 0.082 |
|  | 2000-2001 | 23 | 0.213 | no | 0.809 | 0.017 | 0.024 | 0.04 |
|  | 2002***** | 22 | 0.150 | no | 0.792 | 0.009 | 0.015 | 0.026 |
|  | 2003***** | 26 | 0.455 | no | 0.810 | 0.021 | 0.023 | 0.043 |
|  | 2004-2005***** | 23 | **0.007** | **yes** | **0.690** | **0.001** | **0.000** | **0.001** |
|  | 2006-2008 | 21 | **0.019** | no | **0.708** | **0.000** | **0.001** | **0.002** |
|  | 2009-2012 | 31 | 0.545 | no | **0.666** | **0.000** | **0.000** | **0.000** |
| S101 | 1996-2001† | 30 | 0.326 | no | 0.894 | 0.162 | 0.186 | 0.273 |
|  | 2006-2012† | 26 | 0.180 | no | 0.788 | 0.011 | 0.011 | 0.023 |
|  | 2008-2012 | 85 | 0.248 | no | 0.806 | 0.016 | 0.020 | 0.037 |
| E405 | 2010-2011 | 48 | 0.411 | no | 0.799 | 0.012 | 0.017 | 0.03 |

*Years during which the mange epizootic occurred.

† Malibu Creek State Park subset of S101 population only.

‡ TPM: two-phase model

Table S16. STRUCTURE cluster assignment and posterior probability of correct genetic population assignment of potential migrants in SMMNRA.

| Animal ID | Direction | Structure Analysis Result: Capture Location Cluster ((%) – Cluster Assignment) | Structure Analysis Result: Cluster Location Assignment (Genetic Origin) ((%) – Cluster Assignment) | Posterior Probability of Correct Assignment to Genetic Origin Cluster |
| --- | --- | --- | --- | --- |
| BM016 | S101 -> N101-PM | 0.31 | 0.54 | 0.71 |
| B207 | E405 -> S101 | 0.14 | 0.85 | 0.99 |
| B272 | E405 -> S101 | 0.27 | 0.72 | 0.75 |
| BS25 | E405 -> S101 | 0.17 | 0.82 | 0.97 |
|  |  |  |  |  |

Table S17. The number of alleles per locus per population. Subtle qualitative differences, such as in the number of alleles for immune-linked loci compared with neutral loci, may contribute to the absence of STRUCTURE observed in the immune-linked loci STRUCTURE analysis.

|  | **Neutral loci** | | | | | | | | |  | **Immune-linked loci** | | | | | | |
| --- | --- | --- | --- | --- | --- | --- | --- | --- | --- | --- | --- | --- | --- | --- | --- | --- | --- |
| Populations | FCA  008 | FCA  023 | FCA  026 | FCA  043 | FCA  045 | FCA  077 | FCA  090 | FCA  096 | FCA  132 |  | FLA1 | DRA1 | DRB1 | DRB3 | DRB4 | TLR3 | TLR4 |
| All bobcats | 8 | 6 | 15 | 5 | 8 | 6 | 6 | 8 | 8 |  | 8 | 12 | 4 | 9 | 8 | 9 | 3 |
| E405 | 5 | 4 | 9 | 4 | 3 | 7 | 5 | 5 | 6 |  | 5 | 7 | 4 | 9 | 5 | 4 | 2 |
| S101 | 8 | 5 | 14 | 5 | 7 | 6 | 6 | 7 | 8 |  | 7 | 11 | 4 | 6 | 8 | 9 | 2 |
| N101-B/DM | 5 | 5 | 11 | 5 | 6 | 7 | 5 | 6 | 5 |  | 7 | 12 | 4 | 8 | 8 | 8 | 3 |
| N101-PM | 7 | 6 | 15 | 5 | 8 | 7 | 6 | 8 | 7 |  | 8 | 7 | 4 | 8 | 7 | 6 | 3 |

|  |  |  |  |  |
| --- | --- | --- | --- | --- |
