# Supplementary material for: Disease and freeways drive genetic change in urban bobcat populations
Source: Evol Appl. 2014 Dec 2;8(1):75–92. doi: 10.1111/eva.12226 (PMC4310583; doi:10.1111/eva.12226)
Supplement: Supplementary file 4 [file eva0008-0075-sd4.docx]

**Supporting figure legend**

Figure S1. Results of population structure analyses using immune-linked and neutral loci. N101-B/DM: N101 before/during mange population; N101-PM: N101 post-mange population. (A) Analysis performed with seven immune-linked loci for *K* = 2-5. (B) Analysis performed with six immune-linked loci for *K* = 2-5. DRB1, found to be in linkage disequilibrium with three other immune-linked loci, was excluded from the analysis. (C) Analysis performed with six neutral loci. Less population structure was resolved in comparison with nine neutral loci, although still more structure than resolved with six (B) or seven immune-linked loci (A).

Figure S2. The harvested results of STRUCTURE analyses as plots of mean LnP(K) as a function of K (with standard deviations) and delta K as a function of K. (A) Harvested results of STRUCTURE analysis with nine neutral loci for all bobcats. (B) Harvested results of the N101 two-year survivor STRUCTURE analysis with nine neutral loci. (C) Harvested results of the STRUCTURE analysis with seven immune-linked loci for all bobcats. (D) Harvested results of the STRUCTURE analysis with six immune-linked loci (DRB1 excluded) for all bobcats.
